# Toward functional and structurally complex Frank–Kasper phases *via* creating concavities on supramolecular micelles

**DOI:** 10.1039/d5sc07961f

**Published:** 2026-01-22

**Authors:** Yong-Rui Wang, Jui-Heng Weng, Shing-Jong Huang, Chun-Jen Su, U-Ser Jeng, Po-Ya Chang, Wei-Tsung Chuang, Chien-Lung Wang

**Affiliations:** a Department of Chemistry, National Taiwan University No. 1, Sec. 4, Roosevelt Rd Taipei 10617 Taiwan kclwang@ntu.edu.tw; b National Synchrotron Radiation Research Center 101 Hsin-Ann Road Hsinchu 30092 Taiwan weitsung@nsrrc.org.tw

## Abstract

In supramolecular chemistry, higher structural complexity enables emergent functions in ordered soft matter. To construct structurally complex and functional Frank–Kasper (FK) phases, heterogeneity in supramolecular micelles is introduced by blending rigid aromatic dendrons (Ar_2_) with flexible aliphatic dendrons (D_2_). This approach creates micelles with surface concavities while preserving long-range periodicity of the FK *σ* lattice. The concave domains serve as enzyme-like pockets that accommodate guest molecules and facilitate photodimerization reactions. Structural analyses confirm that these features enhance complexity in hierarchical architecture and enable catalytic performance. This work presents a versatile strategy for designing FK phases that integrate molecular recognition, supramolecular precision, and catalytic function within an ordered framework.

## Abstract 2

Concavity-encoded supramolecular micelles enable structurally complex and functional soft Frank–Kasper phases.

## Graphical abstract

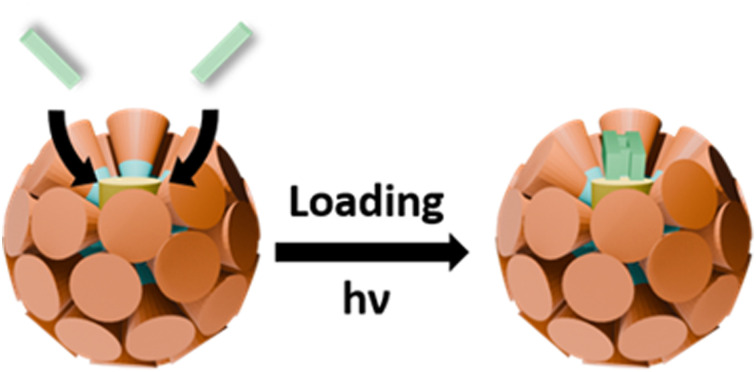

## Introduction

Nature creates self-assembly systems that are precise, complex and dynamic to enable physiological functions that are essential for life.^1^ In comparison to their natural counterparts, artificial supramolecular systems still exhibit relatively limited structural complexity and functionality. To address this gap, researchers often tune two key parameters: multiplicity, which introduces chemical diversity in molecular composition and function, and interconnections, which define the bonding modes and dynamic interactions between components.^2,3^ Modulating these parameters can influence how individual components integrate into higher-order architectures. As this hierarchical integration becomes more sophisticated, the overall organization of the system may change, giving rise to emergent collective properties.^2,3^

One strategy to enhance complexity is the incorporation of aperiodicity within periodic lattices. In block copolymers, factors such as conformational asymmetry,^4–7^ volume asymmetry,^8–10^ compositional asymmetry,^11–15^*etc.* have been applied to create symmetry breaking that leads to structural complexity in the spherical phases.^16^ Blending, in particular, provides a straightforward means of controlling both formation and stability of Frank–Kasper (FK) phases, where uneven interfacial curvature promotes non-spherical and multi-sized micellar motifs.^11,12,17–23^ However, blending-based polydispersity mainly alters micelle size and shape, and therefore offers only limited ways to introduce new structural features. Creating additional levels of complexity—such as spatially defined chemical environments, anisotropic interfaces, or directional binding sites—without disrupting the integration of the original FK lattice remains challenging. For example, although blending block copolymers can effectively shift the phase behavior of mixed systems, the resulting structures often retain a single pre-existing ordered phase, without generating finer structural features or new functional elements within that lattice.^12^ This highlights the difficulty of enhancing structural complexity while maintaining long-range order.

Dendrons and dendrimers offer a distinctive pathway to such complexity and structural features.^24,25^ Their generation-dependent shape evolution,^26–28^ precise molecular design,^26,29–32^ and versatile end-group modifications^27,30,33,34^ enable quasi-equivalent packing that relieves frustration and supports tetrahedrally close-packed FK lattices.^35^ Consequently, dendrons assemble into a remarkable diversity of structures—including cubic,^27,29,30,35,36^ tetragonal,^28,31,35,37,38^ quasicrystalline,^28,37–39^ hollow,^33,34,37^ and even chiral micelles^34,39,40^—surpassing what is typically achievable with block copolymers.^31,37^ In contrast, natural blended systems routinely exploit heterogeneity to generate structural multiplicity and dynamic functionality. Biological membranes, for instance, form compositionally heterogeneous microdomains such as lipid rafts, stabilized by cholesterol–sphingolipid interactions that enhance membrane responsiveness.^41–44^ Likewise, protein binding pockets provide concave hydrophobic cavities that couple structure to function, enabling selective guest recognition and catalysis by pre-organizing substrates and stabilizing transition states.^45–48^ These examples illustrate how blending-derived heterogeneity can simultaneously increase the complexity and functional adaptability of an assembly.

**Scheme 1:**
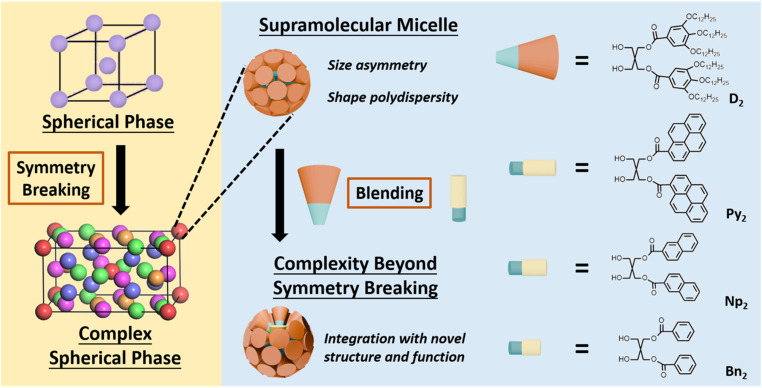
(a) The element of complexity in the current FK phases – symmetry breaking. (b) Illustration of the strategy to further elevate the complexity in the FK phase – introducing heterogeneity and concavity by adding the molecular rafts – Ar_2_ into the supramolecular micelles of D_2_. The right panel shows the chemical structures of D_2_ and the three Ar_2_ molecules, Py_2_, Np_2_, and Bn_2_.

Motivated by these natural design principles—and by the ability of dendrons to introduce controlled heterogeneity—we sought to install designed concavities onto the surface of FK-phase supramolecular micelles. Our goal was to elevate the structural sophistication of the spherical assemblies while preserving the symmetry breaking and long-range integration of the FK lattice. As illustrated in Scheme 1, the diaryl amphiphiles (Ar_2_) are used as molecular rafts that create heterogeneity in supramolecular micelles formed by the amphiphilic di-dendron (D_2_). D_2_ molecule has been synthesized as the intermediate product of the Janus dendrimers for many research.^49–59^ In our previous study, D_2_ has demonstrated the ability to form FK *σ* phase with symmetry breaking.^59^ Since the Ar_2_ molecules are smaller and more rigid than D_2_, blending the Ar_2_ and D_2_ in the supramolecular micelles could create both heterogeneity and concavities in the supramolecular micelles of the FK phase. Catalytic functions mimic the natural hydrophobic pockets may also be added to the complex FK phase as the concavities on top of the Ar_2_ motifs could encapsulate guest molecules. Our characterization data indicated that on top of the symmetry breaking, the presence of the raft-inspired building blocks Ar_2_ in the supramolecular micelles of D_2_ created additional structural features and function to the FK *σ* phase. The overall integration of the synthetic self-assembly system is thus upgraded as the presence of the Ar_2_ advances both hierarchical complexity and functional adaptability of the spherical phase. This innovation aligns with Lehn's vision of progressive complexity,^3^ bridging the gap between structural sophistication and dynamic functionality in artificial self-assembly systems.

## Results and discussion

### Create concavities onto the FK *σ* phase

Schemes S1–S3 illustrate the synthetic routes for D_2_ and Ar_2_. The synthesis routes of these di-dendron molecules are referring to the published literature of dendron self-assembly.^59^ The molecular structures of these compounds were confirmed by ^1^H NMR, ^13^C NMR, and mass spectrometry, as presented in Fig. S2–S15. To incorporate the Ar_2_ into D_2_, both Ar_2_ and D_2_ were co-dissolved in CH_2_Cl_2_. After solvent removal, Ar_2_/D_2_ mixtures with varying molar fractions of Ar_2_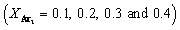 were obtained. The ordered phases of the mixtures were then prepared by heating to 150 °C—above the isotropization temperatures (T_i_) of both D_2_ and Ar_2_ but below their decomposition thresholds (Fig. S16)—to ensure homogeneous mixing in the molten state. The resulting melts were cooled and annealed at 40 °C for 12 hours to facilitate self-assembly.

**Fig. 1:**
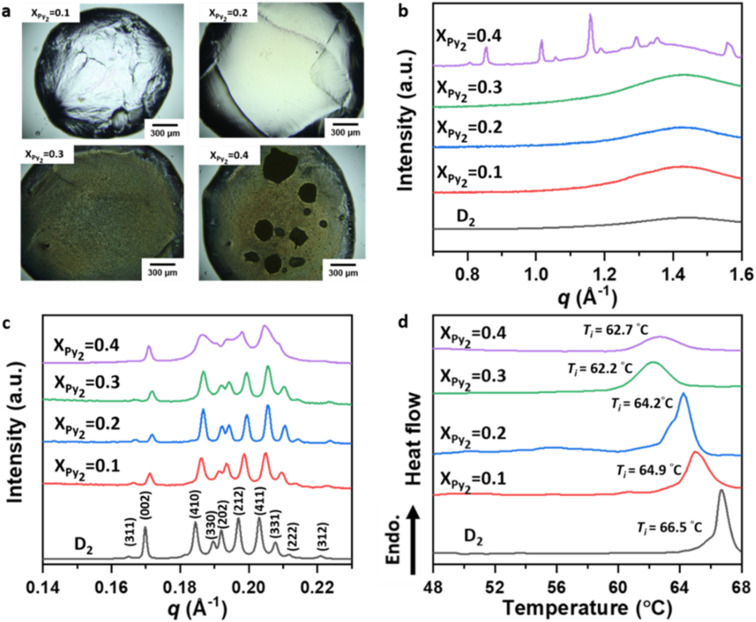
(a) OM micrographs of drop-liquid samples. (b) WAXS and (c) SAXS patterns of the Py_2_/D_2_ mixtures. (d) DSC thermograms of the Py_2_/D_2_ mixtures. Note: (1) The lattice parameters of Py_2_/D_2_ mixtures and the diameter of these supramolecular micelles are shown in Table S2. (2) The DSC parameters of Py_2_/D_2_ mixtures are summarized in Table S3.

To confirm the uniform incorporation of molecular rafts into the ordered phase of D_2_, optical microscopy (OM) was employed for direct observation. As shown in Fig. 1a, macroscopic spherulitic structures were evident in the drop-cast samples of the Py_2_/D_2_ mixtures. With increasing Py_2_ content, the spherulitic morphology became increasingly disrupted. At 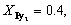 the emergence of dark aggregated domains suggests phase segregation of excess Py_2_. This conclusion is further supported by wide-angle X-ray scattering (WAXS) data (Fig. 1b), where distinct crystalline diffraction peaks attributed to Py_2_ are observed in the 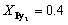 sample. In contrast, WAXS profiles of samples with 
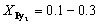
 display no sharp crystalline peaks, but instead feature a broad amorphous halo. This indicates that the spherulitic structures observed *via* OM do not originate from angstrom-scale crystalline ordering. The scattering profiles of varies of Ar_2_ are shown in Fig. S17.

To probe the internal aggregate structures within these spherulites, small-angle X-ray scattering (SAXS) analysis was conducted (Fig. 1c). The SAXS profiles revealed that self-assemble micellization of the Py_2_/D_2_ mixtures crystalizes into the FK *σ* phase. To further clarify how the SAXS data support the micellar assignment and FK *σ* phase formation, the primary and higher-order peaks were analyzed. The peak ratios (*q*/*q*_0_) are consistent with the reflection pattern of the FK *σ* lattice,^60^ and the comprehensive analysis of the *σ*-phase structure and its indexed diffraction peaks is provided in Table S1. The characteristic peaks *q*_410_, *q*_330_, and *q*_002_ were used to calculate the corresponding lattice parameters (*a*, *b*, *c*) by the standard geometric relations of tetragonal phase, shown in Table S2.^38^ Based on these lattice parameters, the diameter of the supramolecular micelles within the FK *σ* lattice (*D*_sphere_) was estimated, also performed in Table S2. These results confirm that the incorporation of Py_2_ does not inhibit the ability of D_2_ to act as a cone-shaped motif and self-assemble into the FK *σ* phase. However, the presence of Py_2_ shifts the diffraction peaks to higher *q* values and broadens them, indicating that the inclusion of rigid, small Py_2_ molecules introduces heterogeneity within the micelles and compresses the *σ*-phase lattice.

The formation of this FK *σ* phase from cone-shaped motifs represents a thermodynamically stable self-assembly, further validated by differential scanning calorimetry (DSC). As shown in Fig. 1d, the T_i_ of the mixtures systematically decreases with increasing Py_2_ content. This phase behavior is consistent with the principle of colligative properties, where the incorporation of solute molecules (Py_2_) lowers the T_i_ of the host component (D_2_).^61^ This behavior also confirms that Py_2_ is well-dispersed within the D_2_-rich ordered domains for compositions with 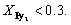 At 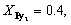 however, the T_i_ does not decrease further, indicating that the excess Py_2_ exceeds the micelle's incorporation capacity and instead undergoes phase separation to form Py_2_-rich domains, as corroborated by both Fig. 1a and b. Additionally, the broadening of all (*hkl*) diffraction peaks in the SAXS profiles supports the DSC results, reinforcing the conclusion that Py_2_ molecules are randomly and uniformly distributed within the supramolecular micelles of D_2_, resulting in both a reduction in T_i_ and broadened diffraction features.

The miscibility of other Ar_2_ variants—Np_2_ and Bn_2_—with D_2_ was also assessed using the same protocol. The results, summarized in Fig. S18 and Tables S4–S9, confirm that both Np_2_ and Bn_2_ exhibit good miscibility with D_2_ up to 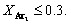 Moreover, increasing size mismatch between Ar_2_ and D_2_ leads to a greater suppression of T_i_ and more significant lattice contraction in the *σ* phase. This trend suggests that smaller molecular rafts introduce greater disruption to the micellar packing order, highlighting the influence of geometric compatibility on structural organization within FK *σ* phases.

The heterogeneity of the Py_2_/D_2_ micelles and the dynamic behavior of Py_2_ within the supramolecular micelles were further investigated by solid-state ^13^C–^1^H heteronuclear correlation (HETCOR) nuclear magnetic resonance (NMR) spectroscopy. For pure D_2_ in the FK *σ* phase, cross-polarization (CP) is relatively inefficient due to the high segmental mobility; therefore, the HETCOR spectrum was acquired using a prolonged CP contact time (t_c_) of 2 ms. In contrast, the HETCOR spectrum of Py_2_ was recorded with a short CP contact time of 200 µs to suppress undesired long-range polarization transfer.

**Fig. 2:**
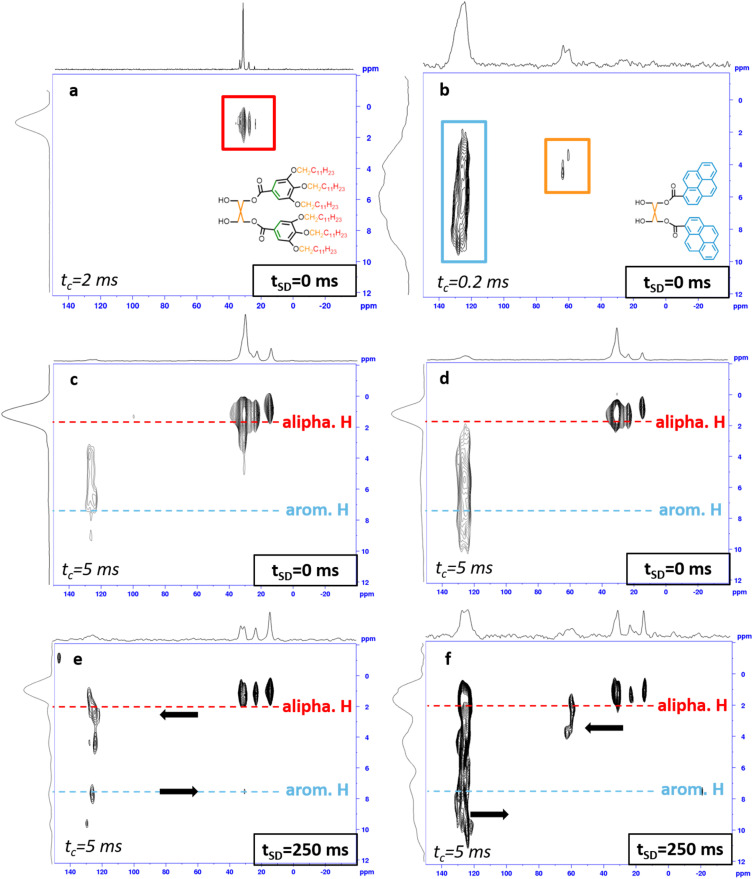
(a) The ssNMR HETCOR spectra (t_SD_ = 0 ms) of pure D_2_, (b) pure Py_2_, (c) Py_2_/D_2_ mixture with 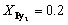 and (d) Py_2_/D_2_ mixture with 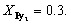 (e) The proton spin-diffusion- incorporated HETCOR spectra of the 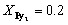 mixture at t_SD_ = 250 ms and (f) the 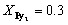 mixture at t_SD_ = 250 ms.

**Scheme 2:**
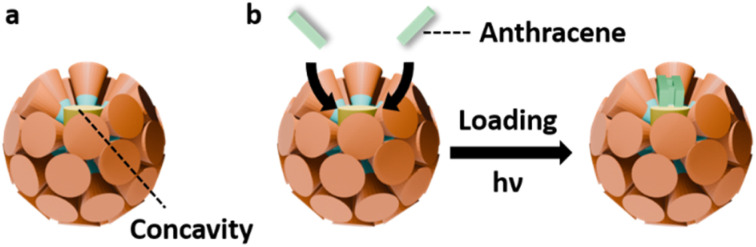
(a) The surface cavity created by Ar_2_ in the concaved supramolecular micelles of the σ(Ar_2_). (b) The photodimerization catalytic process of anthracene within the concaved supramolecular micelle.

As shown in Fig. 2a and b, the characteristic spectral features of D_2_ and Py_2_ are clearly distinguishable. For D_2_, only cross-peaks originating from the dodecyl chains are observed, appearing in the ranges of *δ*_C_ = 10–50 ppm and *δ*_H_ = 0–3 ppm. In contrast, the expected resonances from the O–CH_2_ units (*δ*_C_ ≈ 65–75 ppm),^62^ the pentaerythritol core (*δ*_C_ = 50–65 ppm and *δ*_H_ = 2–5 ppm), and the aromatic moieties (*δ*_C_ = 100–120 ppm and *δ*_H_ = 5–8 ppm) are absent. This behavior can be attributed to their significantly higher mobility, which leads to inefficient ^1^H–^13^C dipolar recoupling under the applied CP conditions, as discussed in detail in Fig. S19. For Py_2_, in addition to the signals originating from the pentaerythritol core, an additional broad resonance at approximately *δ*_C_ ≈ 120 ppm was observed, which can be attributed to the **Py** segments. The aromatic ^1^H signals of Py_2_ are widely distributed and predominantly broadened due to strong residual ^1^H–^1^H dipolar couplings that persist even under MAS conditions, as well as the heterogeneous local environments experienced by the Py_2_ segments within the micelles.^63–65^ For the Py_2_/D_2_ mixtures, the cross-peaks associated with Py_2_ are clearly observed in the HETCOR spectra for samples with 
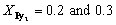
 in the HETCOR spectra, acquired with a prolonged contact time of 5 ms, as shown in Fig. 2c and d. In contrast, the Py_2_ signals in the 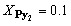 mixture are too weak to be detected (Fig. S20) owing to the low Py_2_ concentration. To gain deeper insight into the location and orientation of the **Py** segments within the micelles, a ^1^H spin-diffusion (PSD) block was incorporated into the HETCOR pulse sequence, rather than further extending the CP contact time, in order to probe long-range intermolecular spatial correlations. This strategy was adopted for three main reasons. First, magnetization decay in the present approach is governed by T_1_ relaxation, whereas an extended CP contact time is limited by T_1_ρ relaxation, and T_1_ is typically much longer than T_1_ρ. Second, ^1^H–^1^H dipolar interactions play a crucial role in long-range intermolecular magnetization transfer; however, the spin-locking RF field ramped linearly from 40 to 50 kHz during CP may partially suppress these dipolar interactions and thus hinder spin diffusion. Third, excessively long CP contact times (>50 ms) may lead to power-handling issues in commercial MAS probeheads. As shown in Fig. 2e and f, at a spin-diffusion time (t_SD_) of 250 ms, magnetization from the ^1^H nuclei of the dodecyl chains is efficiently transferred to the Py_2_ segments. This effective magnetization transfers between the dodecyl chains and the **Py** segments indicates that the **Py** segments of the molecular rafts are primarily located within the hydrophobic shell of the supramolecular micelles, as schematically illustrated in Scheme 2a.

According to the previous work in our group,^59^ in the *σ* phase of D_2_, each supramolecular micelle is constructed with *ca.* 19 cone-like D_2_ molecules. Therefore, at 
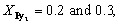
 each supramolecular micelle of Py_2_/D_2_ mixtures has to accommodate *ca.* 4 and 6 Py_2_ molecules, respectively. Combining the ssNMR results in Fig. 2 and the SAXS, WAXS and OM data in Fig. 1, it was found that the presence of Py_2_ creates structural heterogeneity to the supramolecular micelles of D_2_, but the loading capacity of the micelles is limited, as 6 Py_2_ molecules in each micelle is effective enough to create crowded environment that slows down the dynamics of the molecular rafts. The maximum loading capacity of each micelle in the *σ* phase is about 6 Py_2_ molecules, as the higher molar ratio of Py_2_ in the mixture led to the phase-separated Py_2_-rich domains.

A comparable diffusion-based experiment was carried out for the Np_2_/D_2_ system at a molar fraction of 20%, following the same experimental strategy as used for the Py_2_/D_2_ mixtures. As shown in Fig. S21, distinct intermolecular magnetization transfer between the aliphatic region of D_2_ and the aromatic resonances of Np_2_ was observed, indicating efficient spin diffusion between these components. This result demonstrates that replacing Py_2_ with a different aromatic molecular raft (Ar_2_) leads to a similar spatial proximity between the aromatic segments and the dodecyl-chain-rich hydrophobic domains of the D_2_ supramolecular micelles. The Np_2_/D_2_ diffusion data therefore support the generality of aromatic raft incorporation within concaved *σ*-phase micelles, rather than a behavior specific to the Py_2_ system.

### Guest encapsulation and catalytic properties of the σ(Ar_2_)

As D_2_ and Ar_2_ are mismatch in size, incorporating molecular rafts Ar_2_ units into the supramolecular micelles of D_2_ create novel FK *σ* phases, denoted σ(Ar_2_), which contain concave domains as illustrated in Scheme 2a. The indentations on the supramolecular micelles may provide confined environments for guest encapsulation and potential catalytic activation resembling hydrophobic enzyme pockets. To probe this, anthracene (An)—a planar polycyclic aromatic hydrocarbon structurally similar to the Ar segments—was selected as the guest molecule. Meanwhile, the [4 + 4] photodimerization of An molecules under UV light^66^ was applied as the probe for both binding and reactivity within σ(Ar_2_) with the molar ratio 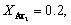 as illustrated in Scheme 2b.

To differentiate the dispersion environments, the An-loaded assemblies are denoted as An@σ for the assemble using simply D_2_ as the host (conventional micelles), and An@σ(Ar_2_) for those using Ar_2_/D_2_ mixtures as the hosts (concaved micelles).

**Fig. 3:**
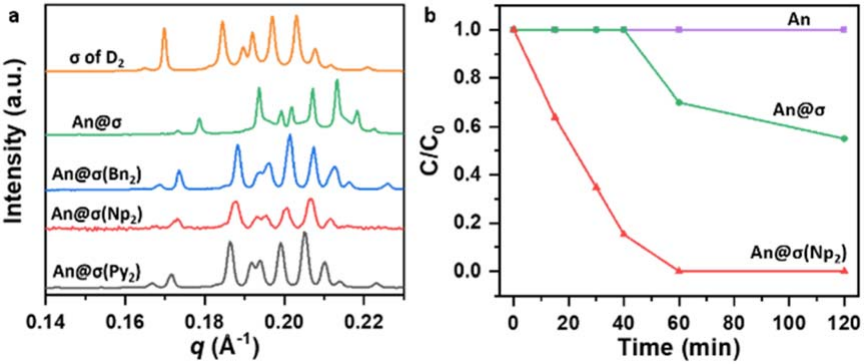
(a) SAXS patterns of **An**@σ(Ar_2_) and **An**@σ systems. (b) Normalized concentration of anthracene (C/C_0_) plotted against irradiation time, monitored by ^1^H NMR spectroscopy. Note: (1) the 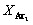 is equal to 0.2 and the molar ratio of the encapsulated **An** and Ar_2_ is 2 : 1 in the **An**@σ(Ar_2_) samples. (2) The indexed diffraction peaks and the lattice parameters and diameter of the supramolecular micelle of **An**-loaded assemblies are shown in Tables S10 and S11. (3) The raw data of ^1^H NMR spectroscopy for the photodimerization are shown in Fig. S24.

SAXS profiles in Fig. 3a shows that guest loading perturbs the lattice of conventional micelles, causing more significant lattice contraction and peak broadening of the *σ* phase of D_2_, as shown in Table S11 and Table S12. The insignificant changes of the FK *σ* framework of σ(Ar_2_) indicate that the pre-organized concavities localize guest molecules and reduce the extent of global lattice distortion. Due to the size differences, the incorporation of Py_2_, Np_2_ and Bn_2_ results in concavities with difference depths in the micelles. Among the variants, σ(Np_2_) provides the optimal balance of geometric complementarity and interaction strength, stably encapsulating 2 – 3 eq. of An without phase-separation (Fig. S22 and S23). Collectively, these results establish that only concavities of suitable depth enable stable guest encapsulation, with σ(Np_2_) standing out by accommodating two An per concavity—precisely the stoichiometry for photodimerization—highlighting the unique role of geometry-tuned concavities in stabilizing and pre-organizing guests.

Since σ(Np_2_) exhibited the most favorable guest encapsulation, we next evaluated its impact on photoreactivity. As shown in Fig. 3b, crystalline **An** dimerized very slowly under UV irradiation (*λ* = 365 nm) due to the rigid packing of the lattice. In contrast, **An** dispersed in conventional micelles (**An**@σ) reached about 45% conversion within 2 h, consistent with the requirement for suitable intermolecular orientation;^67^ the modestly improved conversion can be attributed to the greater molecular mobility in the fluidic micellar environment. Remarkably, in σ(Np_2_) assemblies nearly complete photodimerization occurred within 1 h, demonstrating that the concavities not only stabilize guest encapsulation but also promote effective preorganization of the reactants. Collectively, these results show that concavity-engineered micelles enhance photoreactivity through the combined effects of confinement and spatial preorganization, thereby closely emulating the functional characteristics of enzymatic active sites.

## Conclusions

To exceed complexity of the FK phase and equip functions to the bulk phase supramolecular micelles, in this study, aromatic (Ar_2_) and aliphatic (D_2_) dendrons are blended in controlled ratios to introduce heterogeneity and create surface concavities within the micellar domains of FK *σ* phase. These concavities increase architectural hierarchy through localized asymmetry while preserving the long-range periodicity of the FK *σ* lattice, as evidenced by SAXS and WAXS results. Solid-state NMR reveals that aromatic segments of the Ar_2_ reside in the hydrophobic micellar shell, confirming the formation of chemically distinct, heterogeneous domains. These features generate concavities that act as functional pockets for guest encapsulation without disrupting the FK *σ* lattice. SAXS and WAXS analyses confirm the incorporation of An into the ordered σ(Np_2_) framework, revealing concavities with optimal depth and packing to encapsulate the guest molecules. Building on this structural evidence, time-dependent *ex situ*^1^H NMR demonstrates that such concavities substantially accelerate An photodimerization, as reflected in the C/C_0_ profiles.

Together, these findings establish σ(Np_2_) as the most effective architecture for promoting photoreactivity. More broadly, they highlight a generalizable strategy in which concavity confinement, molecular recognition, and dynamic guest exchange are embedded within a FK *σ* lattice, thereby functionally mimicking enzymatic active sites and advancing Lehn's vision of progressively complex soft materials.

## Author contributions

Conceptualization, data curation, formal analysis: Y. R. W, J. H. W, W. T. C., and C. L. W. Funding acquisition: C. L. W. Investigation: Y. R. W, J. H. W, and S. J. H. Methodology: Y. R. W., S. J. H., and C. L. W. Project administration: Y. R. W., and C. L. W. Resources, software: S. J. H, C. J. S, U. S. J., P. Y. C., W. T. C., and C. L. W. Supervision, validation: W. T. C., and C. L. W. Visualization, writing – original draft: Y. R. W, and C. L. W. Writing – review & editing: Y. R. W, S. J. H., W. T. C and C. L. W.

## Conflicts of interest

There are no conflicts to declare.

## Supplementary Material

SC-OLF-D5SC07961F-s001

## Data Availability

All data supporting the findings of this study, including time-dependent ^1^H NMR spectra, SAXS/WAXS scattering profiles, ssNMR spectra, TGA and DSC thermograms, are provided in the supplementary information (SI). Additional raw data generated and analyzed during the current study are available from the corresponding author upon reasonable request. Supplementary information: the synthetic procedures, ^1^H and ^13^C NMR and MASS spectrum and TGA and DSC thermograms of the D_2_ and Ar_2_, Miller indices, lattice parameters, and S/WAXS patterns of FK *σ* phase of the Ar_2_/D_2_ mixtures, instrumentation of the analytical experiments, ssNMR results and its pulse sequences, characterization and catalytic analysis of An, An@σ, and An@σ(Ar_2_). See DOI: https://doi.org/10.1039/d5sc07961f.
