# Supplementary material for: Toward functional and structurally complex Frank–Kasper phases via creating concavities on supramolecular micelles
Source: Chem Sci. 2026 Jan 22;17(11):5712–9. doi: 10.1039/d5sc07961f (PMC12848762; doi:10.1039/d5sc07961f)
Supplement: SC-017-D5SC07961F-s001 [file SC-017-D5SC07961F-s001.pdf]

Supplementary Information (SI) for Chemical Science.
This journal is © The Royal Society of Chemistry 2026

# Electronic Supporting Information (ESI)

## Toward Functional and Structurally Complex Frank–Kasper Phases via Creating Concavities on Supramolecular Micelles

*Yong-Rui Wang,[a] Jui-Heng Weng,[a] Shing-Jong Huang,[a] Chun-Jen Su,[b] U-Ser Jeng,[b] Po-Ya Chang,[b] Wei-Tsung Chuang,*[b] Chien-Lung Wang*[a]*

[a]*Department of Chemistry, National Taiwan University, No. 1, Sec. 4, Roosevelt Rd, Taipei 10617, Taiwan*

[b]*National Synchrotron Radiation Research Center, 101 Hsin-Ann Road, Hsinchu 30076, Taiwan*

All authors have given approval to the final version of the manuscript.

*** Corresponding Author**

*Email:* kclwang@ntu.edu.tw

*** Corresponding Author**

*Email:* weitsung@nsrrc.org.tw

**Table of Contents**

Experimental Section ......S2
1. Synthetic Procedures of $D_2$ and the Aromatic Derivative Molecules ......S2
2. Analytical Experiments ......S6
3. Preparation of Mixed Micelles ......S9
Characteristic Section ......S11
1. NMR and MS Spectra of Products ......S11
2. Characterization of the $Ar_2/D_2$ Mixtures ......S19
3. Characterization of the An@σ($Ar_2$) ......S26
Reference ......S32

# Experimental Section

## 1. Synthetic Procedures of $D_2$ and the Aromatic Derivative Molecules

*Synthesis of* $\boldsymbol{D_2}$

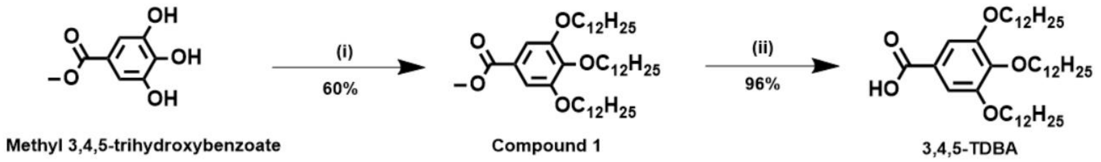


**Scheme S1** The synthetic route of **3,4,5-Tris(dodecyloxy)benzoic acid (3,4,5-TDBA).** (i) 1-Bromododecane ($C_{12}H_{25}Br$), Potassium carbonate ($K_2CO_3$), DMF, 80 °C, 24 h; (ii) Potassium hydroxide (KOH), EtOH/THF/$H_2O$ = 24/2/1, 90 °C, 8 h.

Scheme S1 outlines the synthesis route of the **3,4,5-TDBA**, which serves as the hydrophobic arms of the $\mathbf{D_2}$. Methyl 3,4,5-trihydroxybenzoate (27.54 mmol, 5.10 g) was completely dissolved in DMF (150 mL) at 60 °C before adding $K_2CO_3$ (172.20 mmol, 23.80 g). The mixture was stirred for 2 hours to deprotonate all hydroxyl groups. Subsequently, 1-bromododecane (88.0 mmol, 21.3 mL) was added slowly, and the mixture was stirred at 80 °C for a day. After cooling the reaction mixture to room temperature, is was poured into 750 mL of icy water, followed by suction filtration and recrystallization using acetone, yielding Compound 1 (yield: 60%). The $^1$H-NMR spectra of Compound 1 was shown in Fig. S2. $^1$H NMR (400 MHz, $CDCl_3$) δ 7.25 (s, 2H), 3.99 – 4.02 (m, 6H), 3.89 (s, 3H), 1.72 – 1.85 (m, 6H), 1.46 – 1.49 (m, 6H), 1.26 (m, 48H), 0.88 (t, J = 6.8 Hz, 9H).

Next, Compound 1 (34.82 mmol, 24.00 g) was completely dissolved in an EtOH/THF/$H_2O$ solution (v/v/v = 24/2/1, 400mL) at approximately 60 °C. KOH was added, and the mixture was stirred at 90 °C for 8 hours. After cooling to room temperature, the reaction mixture was poured into 1000 mL of icy water. Diluted

hydrochloric acid (HCl) was added dropwise until the pH of the solution reached 1.0, after which the solution was placed in a refrigerator at 4°C overnight. The resulting precipitate was collected by suction filtration and extract with hexane. The organic layer was dried over anhydrous $MgSO_4$, filtered, and concentrated under reduced pressure using a high-vacuum system to yield a milky white solid that identified as **3,4,5-TDBA** (yield: 96%). The $^1$H-NMR spectra of **3,4,5-TDBA** is shown in Fig. S3. $^1$H NMR (400 MHz, $CDCl_3$) δ 7.30 (s, 2H), 3.99 (q, J = 6.7 Hz, 6H), 1.75 – 1.82 (m, 6H), 1.44 – 1.48 (m, 6H), 1.26 (m, 51H), 0.88 (t, J = 6.7 Hz, 10H).

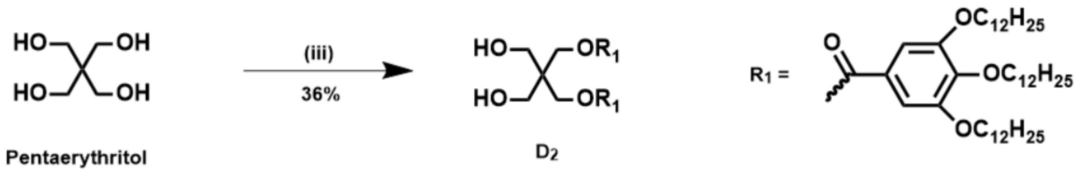


**Scheme S2** The synthetic route of **$D_2$.** (i) 3-[(Ethylimino)methylidene]amino-N,N-dimethylpropan-1-amine (EDC), 4-Dimethylaminopyridine (DMAP), 3,4,5-TDBA, $CH_2Cl_2$/DMF = 3/1, 24 h.

Scheme S2 illustrates the synthesis route of the **$D_2$**. Pentaerythritol (1.10 mmol, 150.0 mg) was completely dissolved in DMF (40 mL) with sonication. $CH_2Cl_2$ (120 mL), **3,4,5-TDBA** (2.20 mmol, 1486 mg), and DMAP (3.30 mmol, 403.8 mg) were added sequentially, and the mixture was stirred in an icy bath. EDC (3.30 mol, 513.0 mg) was then added, and the mixture was stirred for 24 hours to complete the Steglich esterification. The reaction was quenched by adding 400 mL of deionized water, and the product was extracted with $CH_2Cl_2$ three times. The organic layers were wash with water, dried over anhydrous $MgSO_4$, filtered, and concentrated. The crude product was purified by flash column chromatography on silica gel using hexane/ethyl acetate as mobile phase. The purified product was concentrated using a rotary evaporator and a high-vacuum system, followed by washing with methanol to obtain the **$D_2$** (yield: 36%).

The $^{1}H$-NMR, $^{13}C$-NMR and MALDI-TOF MS spectra of **D2** are shown in Fig. S4-Fig. S6. $^{1}H$ NMR (400 MHz, $CDCl_3$) δ7.24 (s, 4H), 4.47 (s, 4H), 4.00 (dt, J = 15.8, 6.4 Hz, 12H), 3.71 (d, J = 6.7 Hz, 4H), 3.01 (t, J = 6.7 Hz, 2H), 1.78 – 1.85 (m, 12H), 1.46 (m, 12H), 1.26 (m, 96H), 0.86 – 0.90 (t, 18H). $^{13}C$ NMR (101 MHz, $CDCl_3$) δ 167.31, 153.12, 143.27, 123.83, 108.45, 73.74, 69.45, 62.97, 46.19, 32.08, 30.50, 29.52, 26.27, 22.84, 14.25. MALDI-TOF $[M+Na]^+$ = 1473.2693 for $C_{91}H_{164}O_{12}$.

*Synthesis of the diaryl dendrons (**Ar2**)*

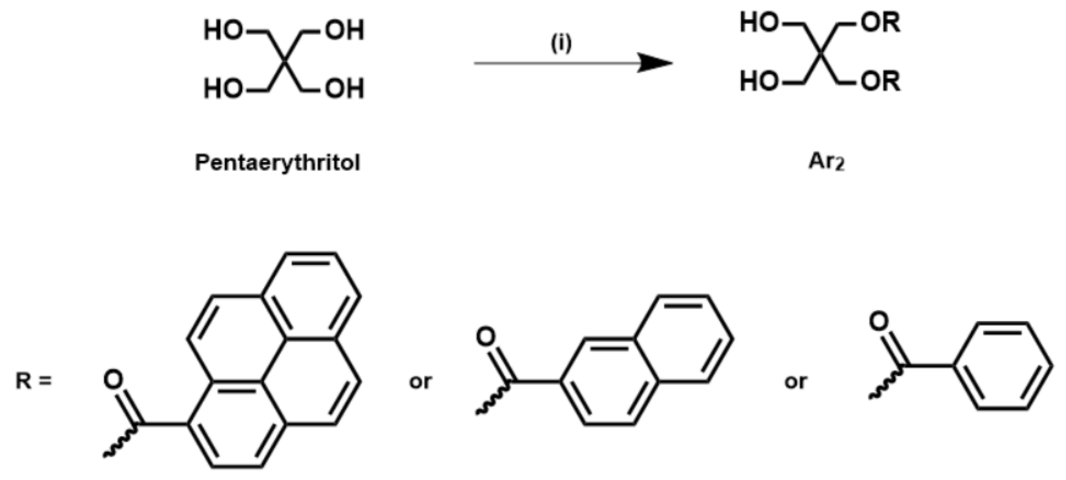


**Scheme S3** The synthetic route of **Ar2** (**Py2**, **Np2**, and **Bn2**). (i) **Py-COOH**, **Np-COOH**, or **Bn-COOH**, EDC, DMAP, $CH_2Cl_2$/DMF = 3/1, 24 h.

Scheme S3 shows the synthesis route of aromatic derivative molecules (**Ar2**), which vary according to the number of aromatic rings in their structures. All synthesis processes are nearly identical to the synthesis of **D2**, as presented in Scheme S2, except for substituting **3,4,5-TDBA** with other acids and adjusting the equivalents of the reactants and purification methods for the Steglich esterification.

For **Py2**, pentaerythritol (1.22 mmol, 166.1 mg) was completely dissolved in DMF (40mL), followed by the sequential addition of $CH_2Cl_2$ (80 mL), Pyrene-1-carboxylic acid (**Py-COOH**, 1.22 mmol, 300.0 mg), and DMAP (1.82 mmol, 223.6 mg). After

stirring, EDC (2.26 mol, 350.4 mg) was added to the reaction mixture under an ice bath, and the stirring was continued for 24 hours. Upon completion of the reaction, the mixture was quenched with water, extracted with $CH_2Cl_2$, washed with water, dehydrated with anhydrous $MgSO_4$, filtered and concentrated. The crude product was then purified by flash column chromatography on silica gel using hexane/ethyl acetate as the mobile phase. The purified product was further concentrated using a rotary evaporator and a high-vacuum system. To enhance purity, reprecipitation was performed by dissolving the crude product in $CH_2Cl_2$, and slowly adding it dropwise into hexane. The final product was collected by suction filtration, followed by solvent removal under high vacuum, yielding **Py2** (yield: 20%). The $^1$H-NMR, $^{13}$C-NMR and ESI-MS spectra of **Py2** are shown in Fig. S7-Fig. S9. $^1$H NMR (400 MHz, $CDCl_3$) δ 9.21 (d, J = 9.4 Hz, 2H), 8.57 (d, J = 8.2 Hz, 2H), 8.26 – 7.89 (m, 14H), 4.79 (s, 4H), 4.00 (d, J = 6.4 Hz, 4H), 2.98 (t, J = 6.4 Hz, 2H). $^{13}$C NMR (101 MHz, $CDCl_3$) δ 168.51, 134.67, 131.48, 131.00, 130.34, 129.93, 129.80, 128.46, 127.07, 126.56, 126.43, 124.70, 124.11, 122.49, 64.10, 63.54, 45.91. ESI-MS $[M+H]^+$ = 593.1933 for $C_{39}H_{28}O_6$. Similar as **Py2**, the synthesis procedures for **Np2** and **Bn2** differ only in the equivalents of the reactants used. For **Np2**, pentaerythritol (1.10 mmol, 150.0 mg) was completely dissolved in DMF (40 mL), followed by the sequential addition of $CH_2Cl_2$ (120 mL), 2-Naphthoic acid (**Np-COOH,** 2.20 mmol, 379.4 mg), DMAP (3.30 mmol, 403.8 mg), and EDC (3.30 mmol, 513.0 mg) under an ice bath for the Steglich esterification. The post-processing steps, as described for **Py2**, were followed, yielding **Np2** (yield: 25%). The $^1$H-NMR, $^{13}$C-NMR and ESI-MS spectra of **Np2** are presented in Fig. S10-Fig. S12. $^1$H NMR (400 MHz, $CDCl_3$) δ 8.61 (s, 2H), 8.05 (d, *J* = 8.4 Hz, 2H), 7.89 (dd, *J* = 17.7, 8.1 Hz, 6H), 7.63 – 7.51 (m, 4H), 4.63 (s, 4H), 3.85 (d, *J* = 6.7 Hz, 4H), 2.96 (t, *J* = 6.7 Hz, 2H). $^{13}$C NMR (101 MHz, $CDCl_3$) δ 167.49, 135.83, 132.53, 131.59, 129.53, 128.68, 127.89, 126.93, 126.72, 125.20, 63.63, 62.99, 45.92. ESI-MS $[M+Na]^+$ = 467.1451 for

$C_{27}H_{24}O_6$.

For **Bn2**, pentaerythritol (1.10 mmol, 150.0 mg) was completely dissolved in DMF (40 mL), followed by sequentially addition of $CH_2Cl_2$ (120 mL), benzoic acid (**Bn-COOH,** 2.20 mmol, 269.1 mg), DMAP (3.30 mmol, 403.8 mg), and EDC (3.30 mmol, 513 mg) under an ice bath for the Steglich esterification. Similarly, the post-processing steps described for **Py2** and **Np2** were applied, yielding **Bn2** (yield: 30%). The $^1$H-NMR, $^{13}$C-NMR and ESI-MS spectra of **Bn2** are shown in Fig. S13-Fig. S15. $^1$H NMR (400 MHz, $CDCl_3$) δ 8.04 (d, $J$ = 7.1 Hz, 4H), 7.59 (t, $J$ = 7.3 Hz, 2H), 7.45 (t, $J$ = 7.8 Hz, 4H), 4.52 (s, 4H), 3.77 (d, $J$ = 6.6 Hz, 4H), 2.84 (t, $J$ = 6.6 Hz, 2H). $^{13}$C NMR (101 MHz, $CDCl_3$) δ 167.27, 133.63, 129.91, 129.56, 128.70, 63.37, 63.01, 45.83. ESI-MS $[M+Na]^+$ = 367.1152 for $C_{19}H_{20}O_6$.

## 2. Analytical Experiments

**Materials and Instrumentation.** $^1$H and $^{13}$C nuclear magnetic resonance (NMR) spectra were obtained using a Bruker AVIII HD 400 NMR spectrometer (400 MHz for $^1$H and 101 MHz for $^{13}$C) with $CDCl_3$ as the deuterated solvent. Measurements were conducted at room temperature to determine the molecular structures. The Matrix-assisted laser desorption/ionization mass spectrometry (MALDI-MS) and the electrospray ionization mass spectrometry (ESI-MS) were performed on a time-of-flight (TOF) instrument to determine the molecular weight of the synthetic compounds. All solid-state nuclear magnetic resonance (ssNMR) spectra were recorded on a Bruker AVIII-600 WB spectrometer equipped with a 3.2 mm double-resonance magic-angle-spinning (MAS) probehead. The sample spinning rate was set to 16 kHz. The standard HETCOR pulse sequence is illustrated in Scheme S4a. A 90° pulse of 5 μs was applied to transfer the $^1$H magnetization to the *xy* plane for $t_1$ evolution, followed by polarization transfer to $^{13}$C via cross polarization (CP). The signal was subsequently detected during

$t_2$ under two-pulse phase modulation (TPPM) $^1$H decoupling with an RF field strength of 100 kHz. For pure **D2** in a Lamellar/σ (Lam/σ) coexistence state and **Py2**, a short CP contact time of 200 μs was employed to predominantly probe single-bond correlations. In contrast, for pure **D2** in the σ phase, the CP efficiency was reduced due to high molecular mobility; therefore, a longer contact time of 2 ms was used to enhance CP polarization transfer. For the **Py2/D2** mixtures, an extended CP contact time of 5 ms was applied. To investigate the spatial arrangement of **Py** segments in the **Py2/D2** mixtures, a $^1$H spin-diffusion block was further incorporated into the HETCOR pulse sequence, as shown in Scheme S4b. The recycle delay was set to 2 s.

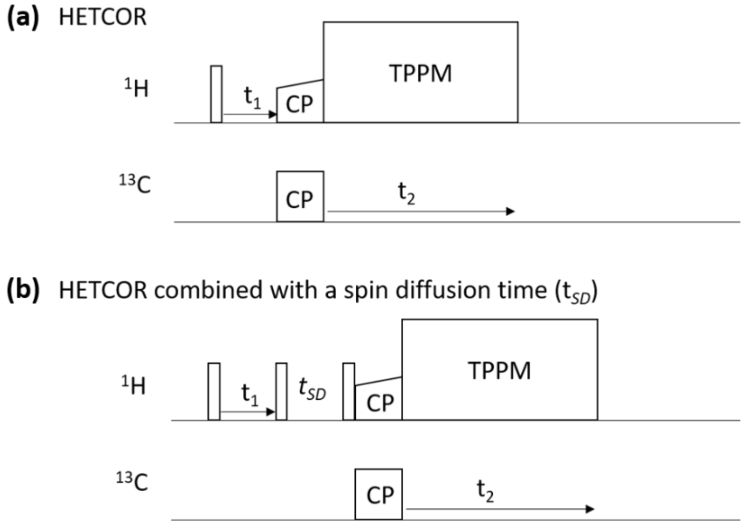


**Scheme S4** (a) Pulse sequences of the HETCOR and (b) the $^1$H spin-diffusion incorporated HETCOR.

**Polarized Optical Microscopy (POM).** The Leica DM270 Polarized Optical Microscope instrument equipped with two polarizers was used for the POM observation. The samples were prepared by placing them on a glass slide substrate, and the polarizers were removed for the OM observation since the FK phases of the mixtures are isotropic, which have no birefringence.

**Thermostability analysis.** Thermogravimetric Analyzer (TGA) analysis was conducted at the Precious Instrument Center of National Taiwan University, using a TA Q500 instrument. The decomposition temperature ($T_d$) of the sample was measured to ensure that the sample would not decompose due to heat during subsequent differential scanning calorimetry (DSC) analysis. The decomposition temperature is defined as the temperature at which the sample experiences a 5% weight loss. Approximately 1–3 mg of the sample was prepared and scanned from 25°C to 600°C at a heating rate of 5°C/min, with nitrogen used as the carrier gas. The DSC analysis was carried out with the Perkin Elmer Pyris Diamond DSC Differential Scanning Calorimeter instrument, operated entirely under a nitrogen atmosphere. Approximately 1-3 mg of the sample is sealed in a Tzero Aluminum Pan with a Lid, while an empty pan serves as the reference. The process involves holding at -20 °C for 1 minute, heating from -20 °C to 150 °C at 10 °C/min, holding at 150 °C for 1 minute to make disordered $\mathbf{Ar_2}$ and $\mathbf{D_2}$ well-mixing, then cooling from 150 °C to -20 °C at 10 °C/min, holding at -20 °C for 1 minute. This cycle is repeated two times, and the $T_i$ of mixtures were measured at the 2$^{nd}$ heating process.

**Small/Wide-angle X-ray scattering (S/WAXS).** Small- and wide-angle X-ray scattering measurements were performed at the Taiwan National Synchrotron Radiation Research Center (NSRRC) on the TPS BL 13A beamline. The synchrotron light source operated at 5.6–25 keV, with an X-ray wavelength of 0.8265 Å and a beam focus size of 400 × 200 μm$^2$. The scattering vector (q) ranged from 0.004 to 0.45 Å$^{-1}$ for SAXS and 0.4 to 1.7 Å$^{-1}$ for WAXS. Approximately 1 mg of each sample was wrapped in two layers of heat-resistant Kapton tape for measurement.

## 3. Preparation of Mixed Micelles

The binary mixtures of **$Ar_2$** and **$D_2$** were prepared as follows: Solid **$D_2$** and **$Ar_2$** were each dissolved in dichloromethane ($CH_2Cl_2$). Using a micropipette, the solutions were combined in appropriate volumes to achieve **$D_2$**-rich mixtures with **$Ar_2$** molar fractions of $\boldsymbol{X_{Ar_2}}$= 0.1, 0.2, 0.3, and 0.4. The resulting mixtures were concentrated using a rotary evaporator to remove the bulk solvent, followed by further drying under vacuum to eliminate residual $CH_2Cl_2$, yielding solid **$Ar_2$/$D_2$** samples.

To promote mixing, the solid mixtures were heated to 150 °C—above the isotropization temperatures ($T_i$) of both **$D_2$** and **$Ar_2$** but below their decomposition thresholds—to ensure homogeneous mixing in the molten state. The melts were then cooled and annealed at 40 °C for 12 h to allow the formation of ordered micellar phases.

To verify that the supramolecular assemblies were fully disrupted at 150 °C prior to annealing, temperature-dependent SAXS measurements were performed on all **$Ar_2$/$D_2$** mixtures over a wide temperature range. Upon heating, the characteristic diffraction features associated with ordered micellar phases progressively vanished, and only a broad amorphous halo was observed above approximately 65 °C. No scattering features of the ordered phase was detected up to 150 °C, confirming that the self-assembled structures were completely isotropized under these conditions, as shown in Fig. S1.

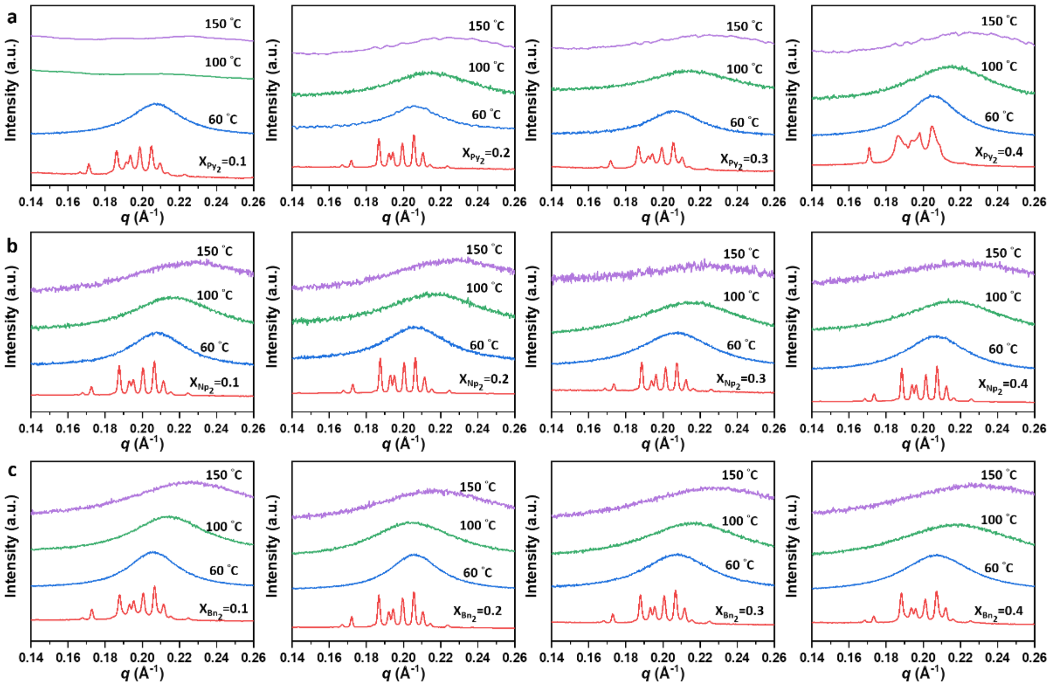

**Fig. S1** The temperature-dependent SAXS profiles of (a) **Py2/D2**, (b) **Np2/D2**, and (c) **Bn2/D2** mixtures at different $X_{Ar_2}$.

For the preparation of anthracene (**An**)-loaded micelles, **An** crystals were dissolved in $CH_2Cl_2$ at the desired concentration. Using a micropipette, a defined volume of the **An** solution was added to the solid **σ(Ar2)** sample. After solvent evaporation under ambient conditions, the resulting samples contained encapsulated **An** molecules within the **σ(Ar2)** micelles.

# Characteristic Section

## 1. NMR and MS Spectra of Products

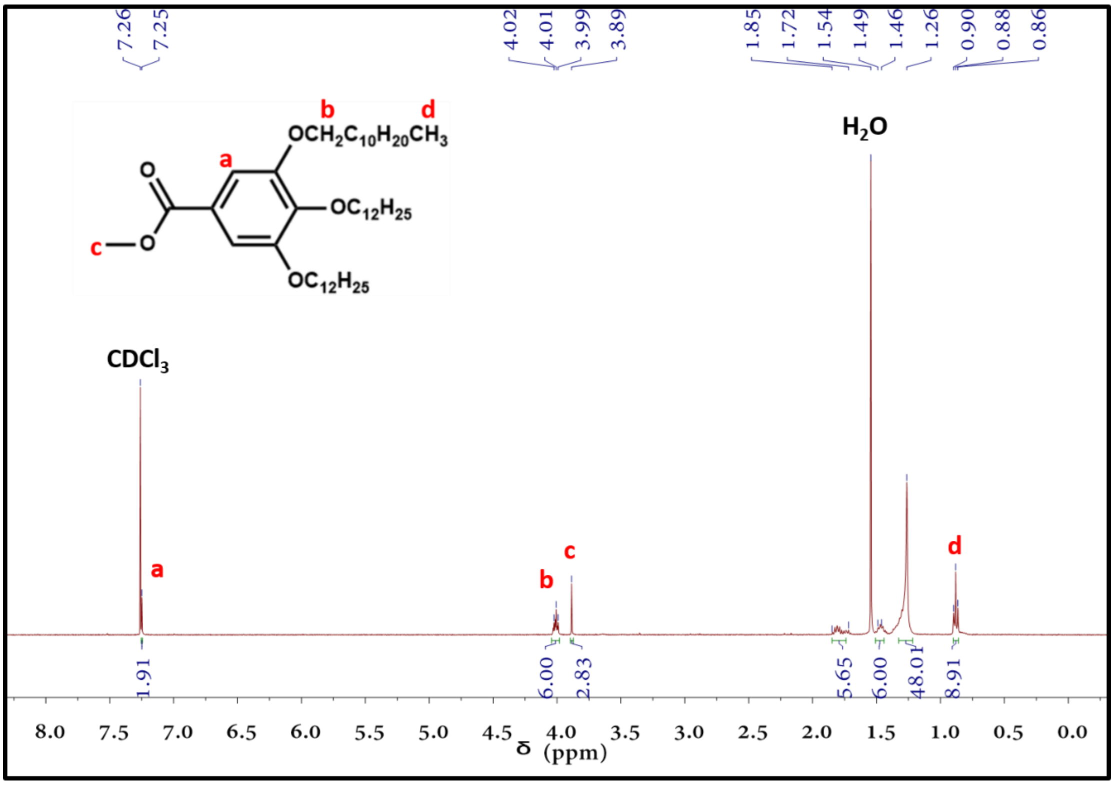


**Fig. S2** $^1$H NMR spectrum of Compound 1 (400 MHz in $CDCl_3$).

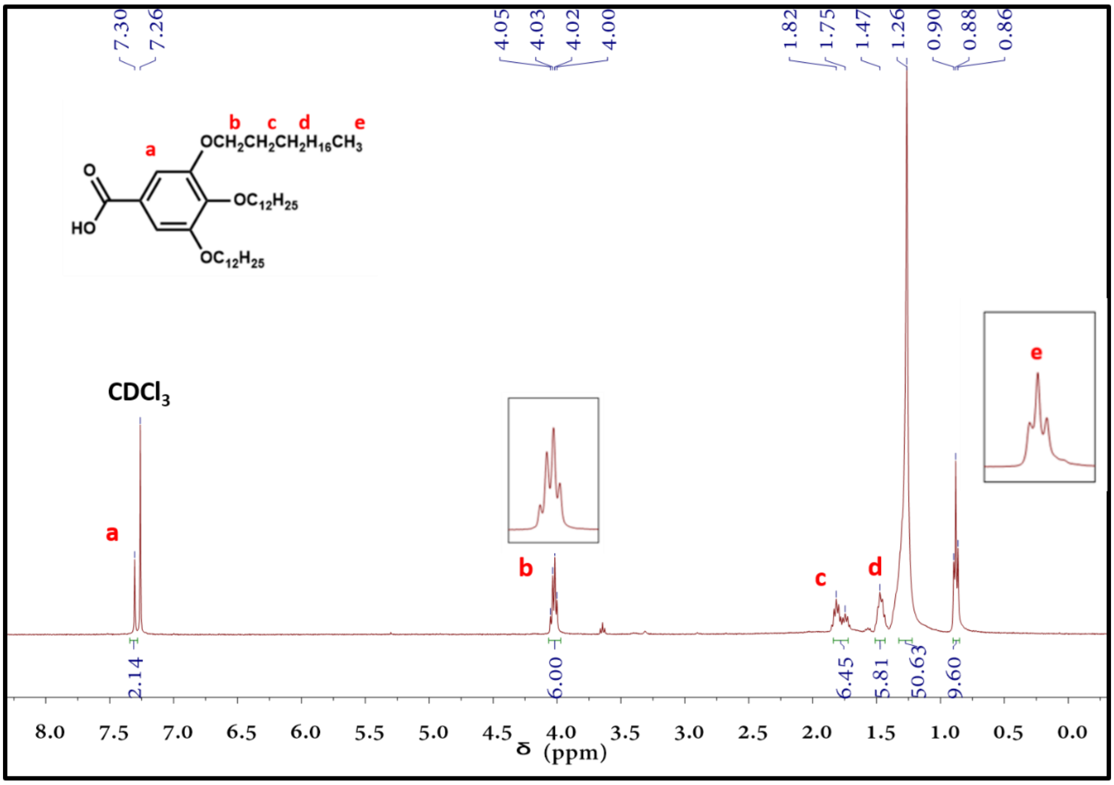


**Fig. S3** $^1$H NMR spectrum of **3,4,5-TDBA** (400 MHz in $CDCl_3$).

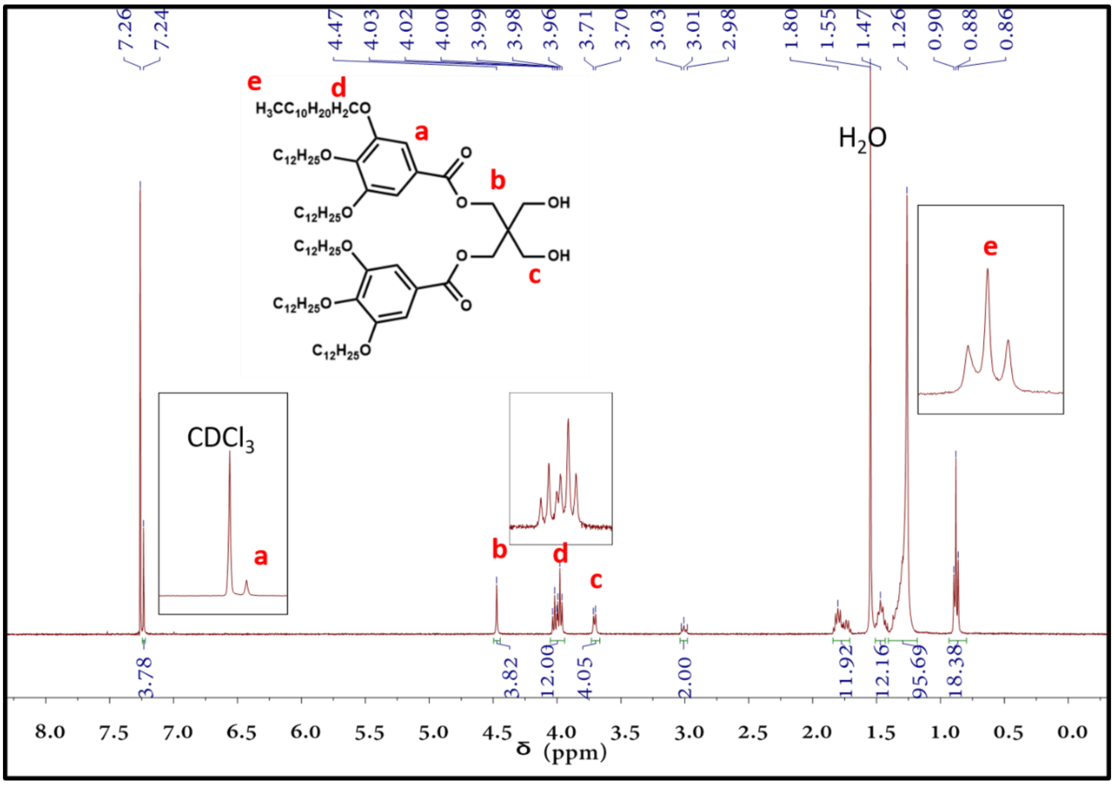


**Fig. S4** $^1$H NMR spectrum of **$D_2$** (400 MHz in $CDCl_3$).

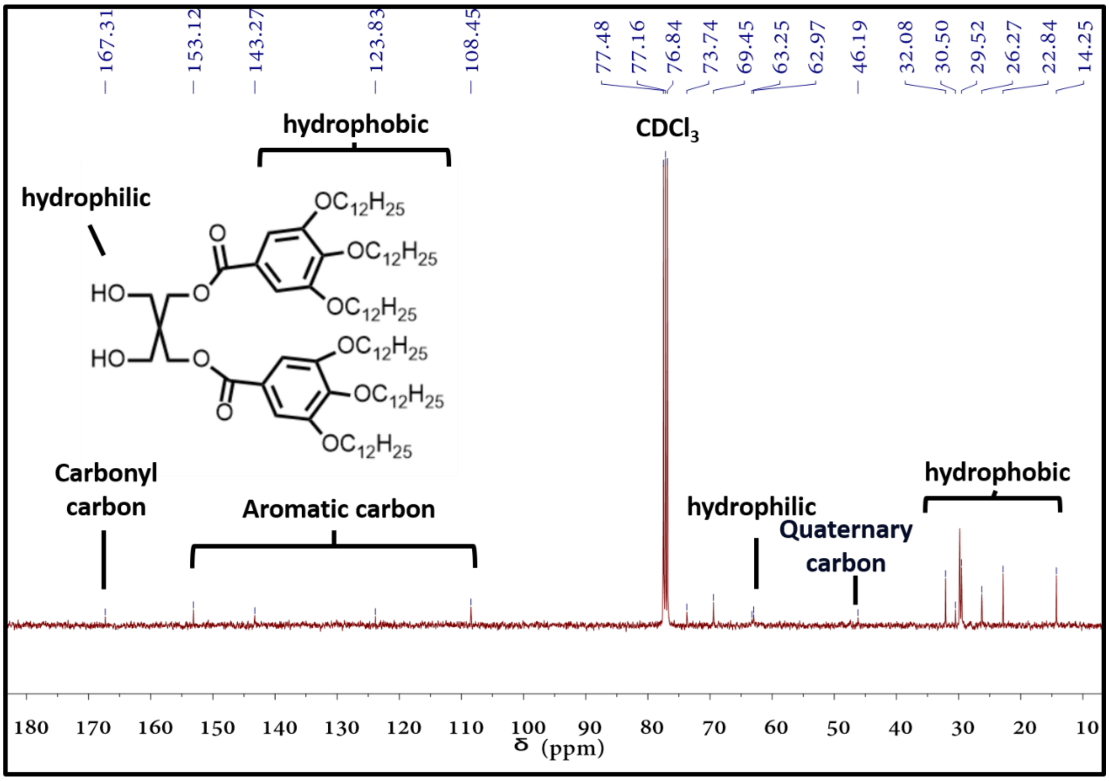


**Fig. S5** $^{13}$C NMR spectrum of **D$_2$** (101 MHz in $CDCl_3$).

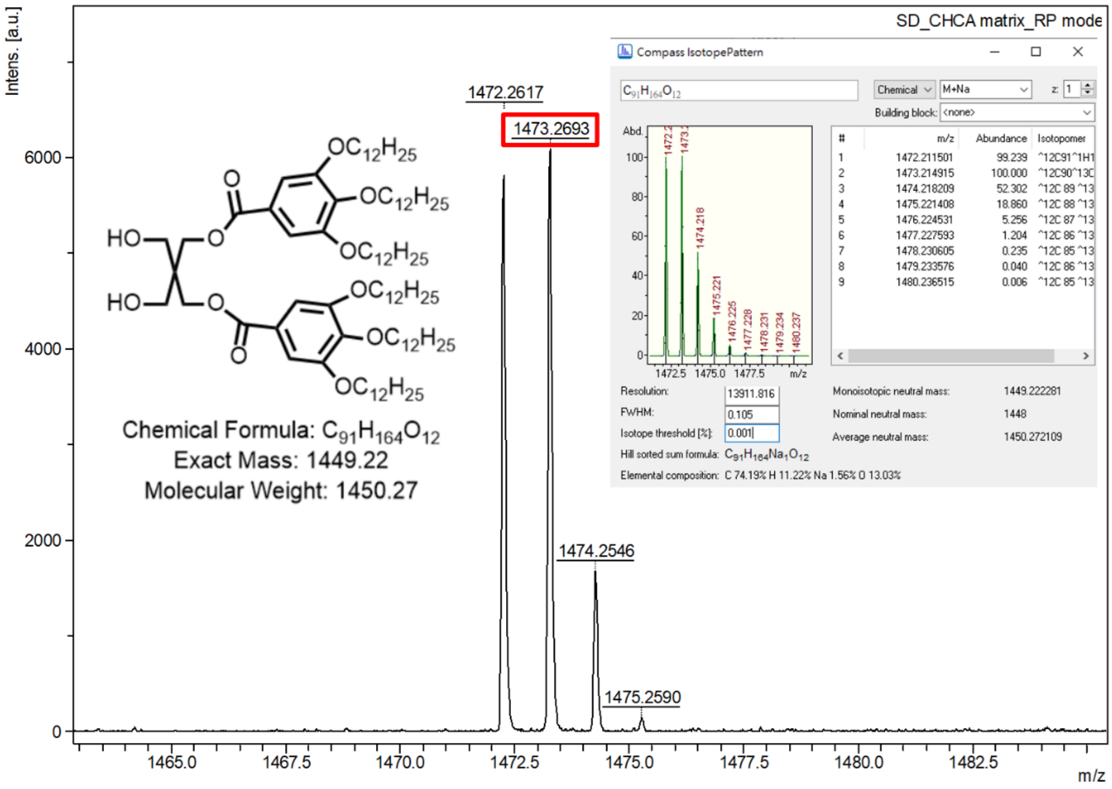


**Fig. S6** MALDI-TOF MS spectrum of **D$_2$** (in CHCA matrix).

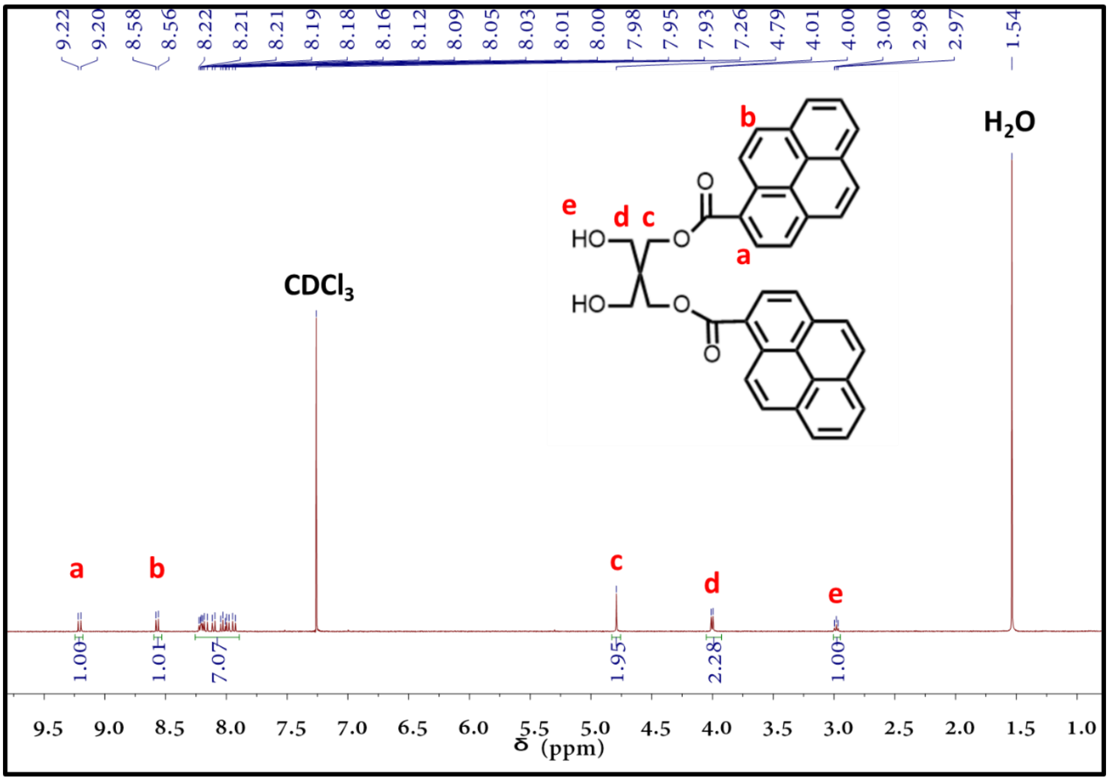


**Fig. S7** $^{1}$H NMR spectrum of **Py2** (400 MHz in $CDCl_3$).

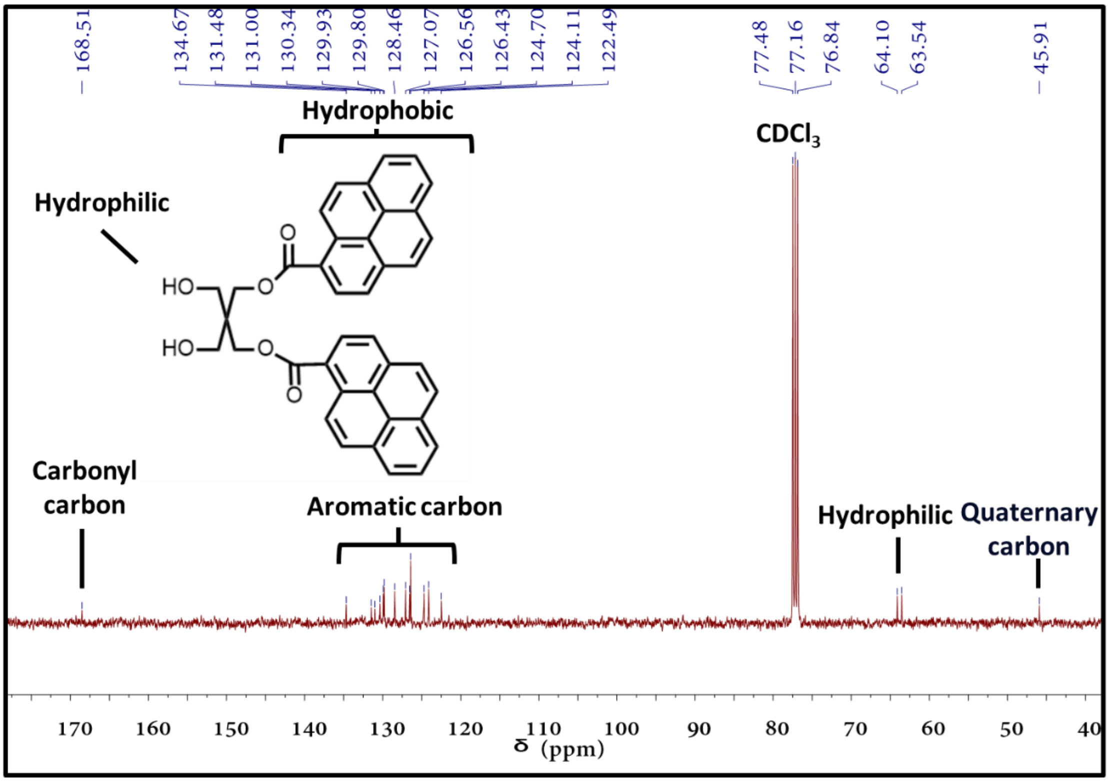


**Fig. S8** $^{13}$C NMR spectrum of **Py2** (101 MHz in $CDCl_3$).

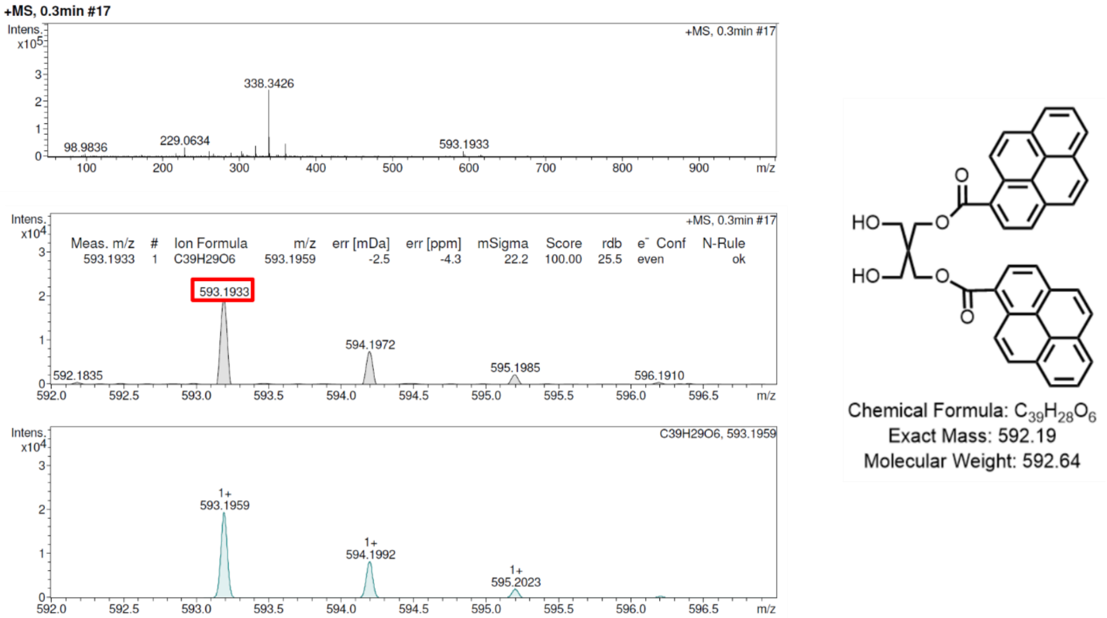


**Fig. S9** ESI-MS spectra of **Py2**.

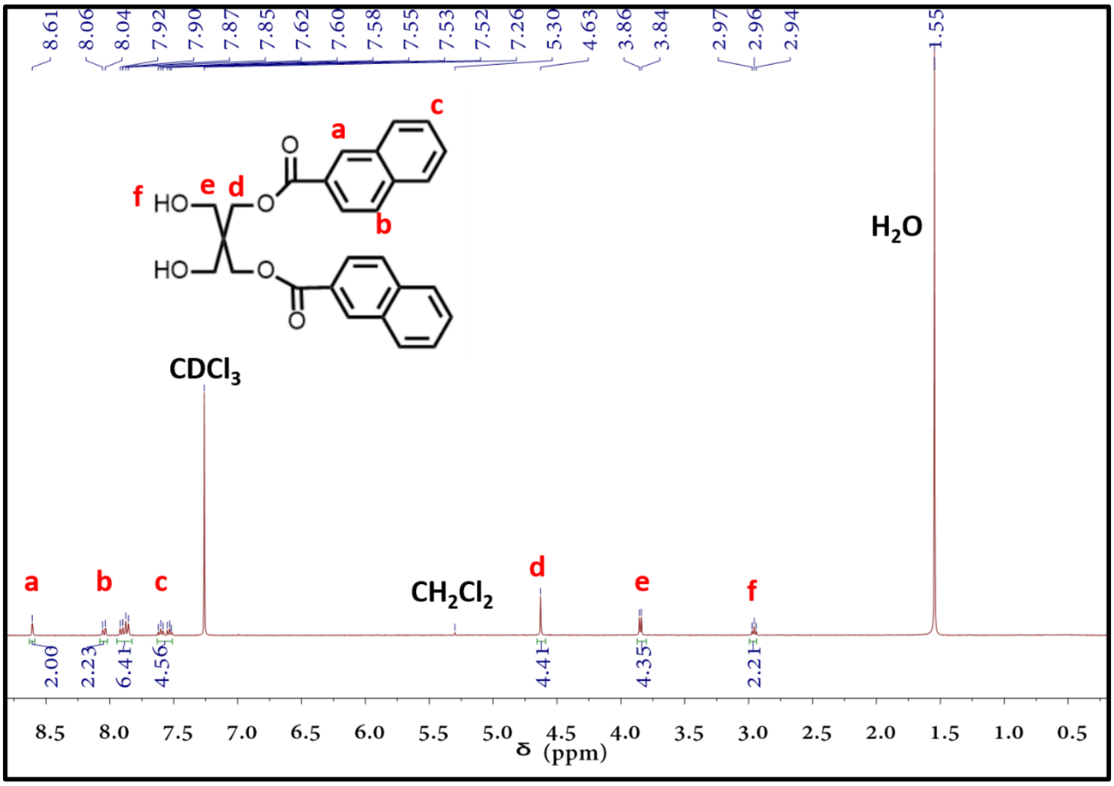


**Fig. S10** $^{1}$H NMR spectrum of **Np2** (400 MHz in $CDCl_3$).

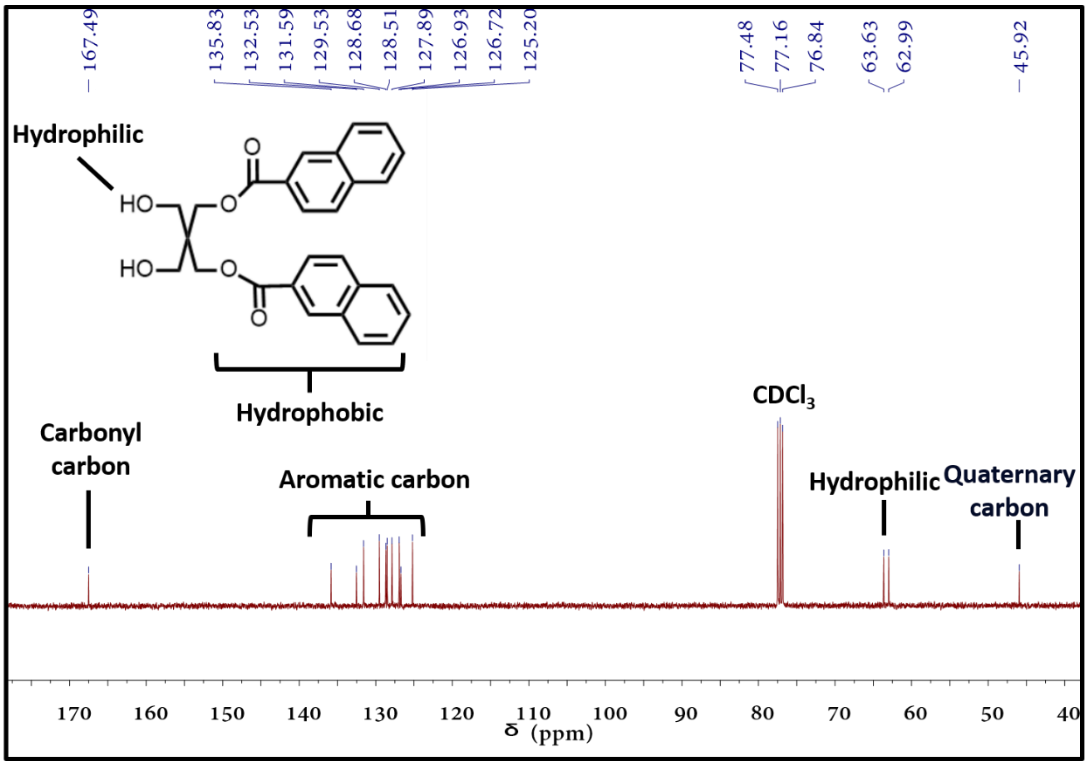


**Fig. S11** $^{13}$C NMR spectrum of **Np2** (101 MHz in $CDCl_3$).

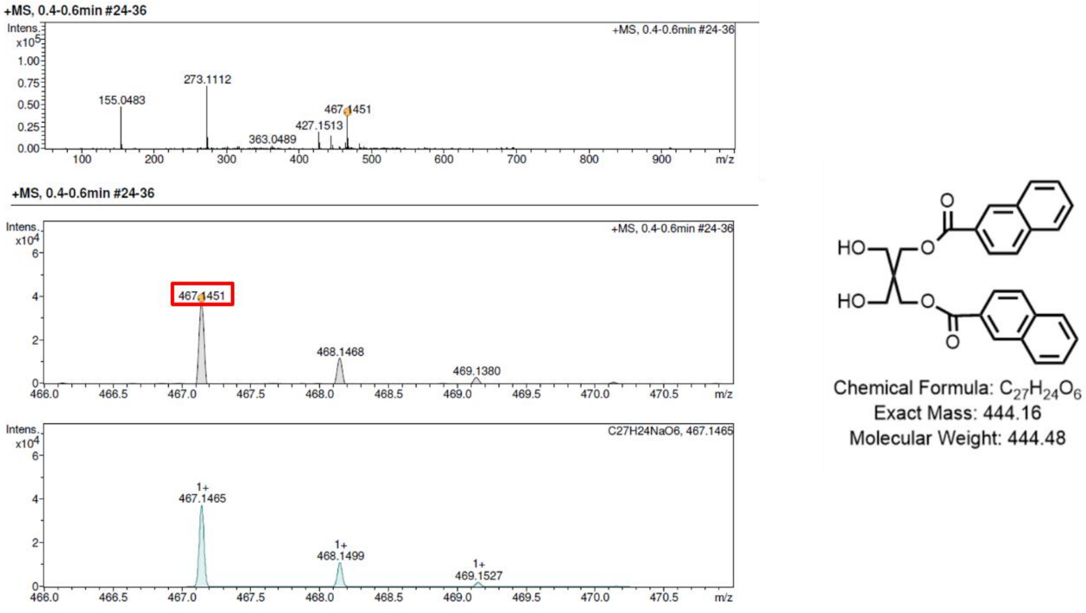


**Fig. S12** ESI-MS spectra of **Np2**.

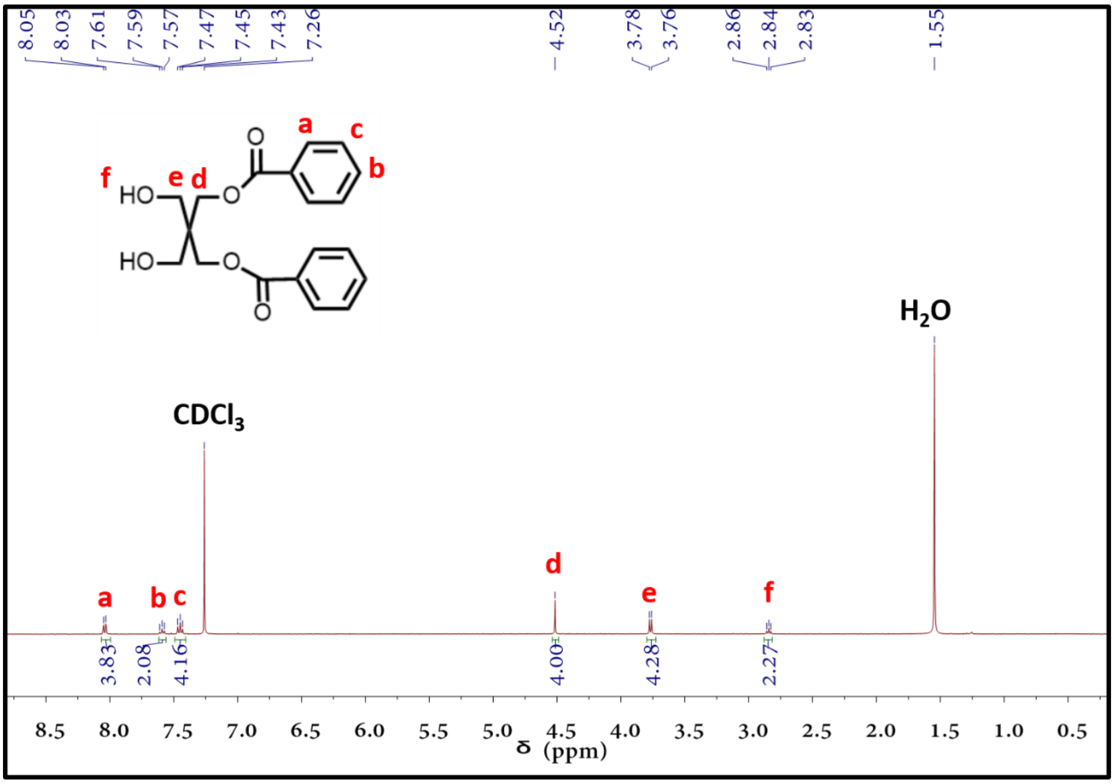


**Fig. S13** $^{1}$H NMR spectrum of **Bn2** (400 MHz in CDCl3).

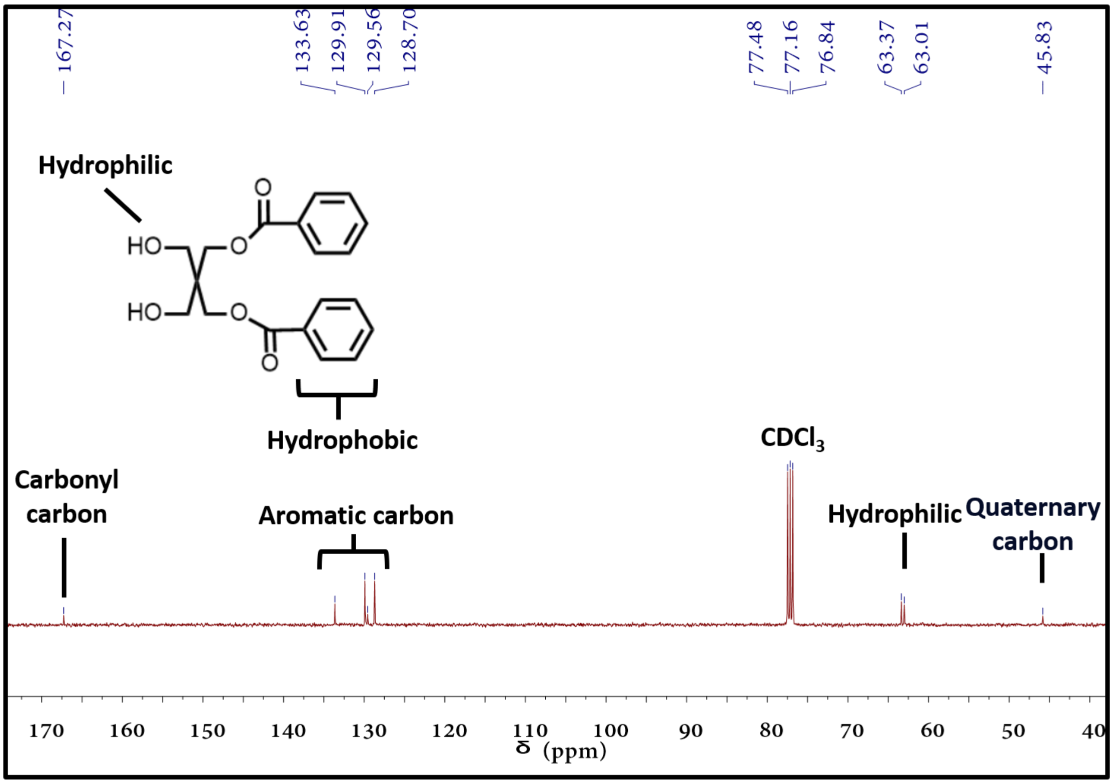


**Fig. S14** $^{13}$C NMR spectrum of **Bn2** (101 MHz in CDCl3).

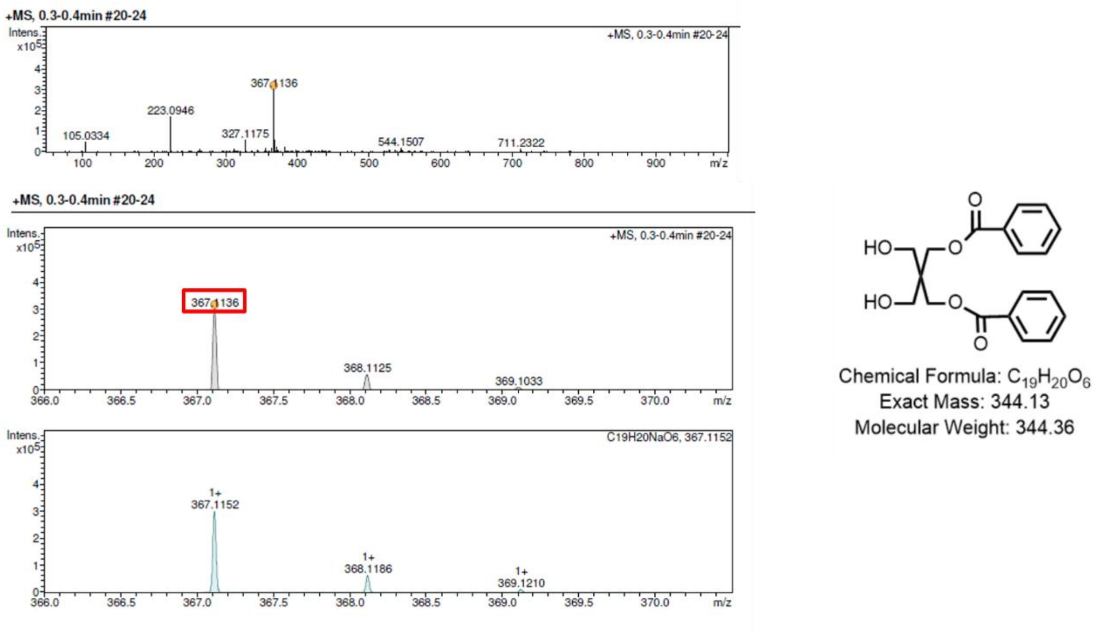


**Fig. S15** ESI-MS spectra of **Bn$_2$**.

## 2. Characterization of the $Ar_2/D_2$ Mixtures

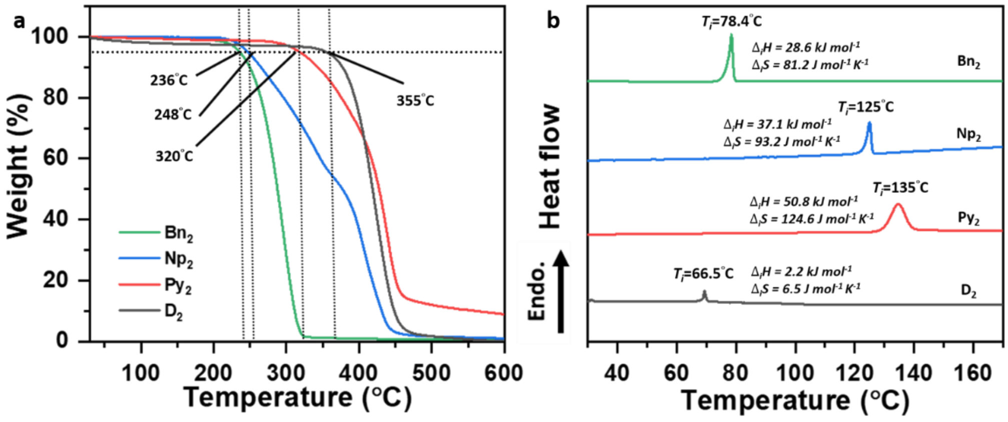


**Fig. S16** The (a) TGA and (b) DSC thermograms of **$D_2$**, **$Py_2$**, **$Np_2$** and **$Bn_2$**.

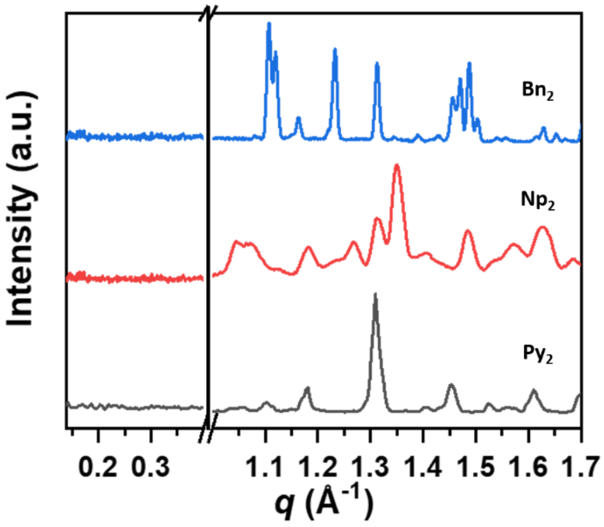


**Fig. S17** The SAXS and WAXS patterns of **$Ar_2$**.

**Table S1** List of the $q$ ratio of the $\boldsymbol{\sigma}$ phase in the literature ($\boldsymbol{q_{lit}}$)[1] and the experimental $\boldsymbol{q_{exp}}$ for the FK σ phase of the **Py2/D2** mixtures. $\boldsymbol{q}$ **ratio** $= \boldsymbol{q}/\boldsymbol{q_0}$, where $\boldsymbol{q_0}$ represents scattering position of the $q_{310}$ diffraction peak.

| Miller index | $\boldsymbol{X_{Py_2}}$ = 0.1 | $\boldsymbol{X_{Py_2}}$ = 0.2 | $\boldsymbol{X_{Py_2}}$ = 0.3 | $\boldsymbol{X_{Py_2}}$ = 0.4 | $\boldsymbol{X_{Py_2}}$ = 0.1 | $\boldsymbol{X_{Py_2}}$ = 0.2 | $\boldsymbol{X_{Py_2}}$ = 0.3 | $\boldsymbol{X_{Py_2}}$ = 0.4 | $q_{lit}$ ratio |
|---|---|---|---|---|---|---|---|---|---|
| | $q_{exp}$ | | | | $q_{exp}$ ratio | | | | |
| (310) | 0.1429 | 0.1429 | 0.1433 | 0.1444 | 1.00 | 1.00 | 1.00 | 1.00 | 1.00 |
| (311) | 0.1664 | 0.1672 | 0.1672 | 0.1674 | 1.16 | 1.17 | 1.17 | 1.16 | 1.17 |
| (002) | 0.1712 | 0.1720 | 0.1720 | 0.1708 | 1.20 | 1.20 | 1.20 | 1.18 | 1.20 |
| (410) | 0.1863 | 0.1867 | 0.1867 | 0.1864 | 1.30 | 1.31 | 1.30 | 1.29 | 1.30 |
| (330) | 0.1915 | 0.1923 | 0.1919 | 0.1906 | 1.34 | 1.34 | 1.34 | 1.32 | 1.34 |
| (202) | 0.1935 | 0.1943 | 0.1943 | 0.1940 | 1.35 | 1.36 | 1.36 | 1.34 | 1.36 |
| (212) | 0.1987 | 0.1994 | 0.1994 | 0.1981 | 1.39 | 1.39 | 1.39 | 1.37 | 1.39 |
| (411) | 0.2050 | 0.2054 | 0.2054 | 0.2046 | 1.43 | 1.44 | 1.43 | 1.42 | 1.44 |
| (331) | 0.2094 | 0.2106 | 0.2102 | 0.2087 | 1.47 | 1.47 | 1.47 | 1.45 | 1.47 |
| (222) | 0.2134 | 0.2142 | 0.2142 | 0.2131 | 1.49 | 1.50 | 1.49 | 1.48 | 1.50 |
| (312) | 0.2237 | 0.2237 | 0.2233 | 0.2220 | 1.56 | 1.56 | 1.56 | 1.54 | 1.56 |

**Table S2** List of the lattice parameters (a, b, c) and the diameter of supramolecular micelles ($D_{sphere}$) for the FK σ phase of the **Py2/D2** mixtures.

| | $\boldsymbol{X_{Py_2}}$ = 0.1 | $\boldsymbol{X_{Py_2}}$ = 0.2 | $\boldsymbol{X_{Py_2}}$ = 0.3 | $\boldsymbol{X_{Py_2}}$ = 0.4 |
|---|---|---|---|---|
| a (Å) | 139.0 | 138.6 | 138.6 | 137.8 |
| b (Å) | 139.0 | 138.6 | 138.6 | 137.8 |
| c (Å) | 73.4 | 73.0 | 73.0 | 73.5 |
| $D_{sphere}$ (Å) | 44.9 | 44.7 | 44.7 | 44.6 |

The calculation of the lattice parameters and supramolecular micelle's diameter are referring to the literature,[2] which is shown in Eq. S1 - Eq. S3 below:

Lattice parameters for FK σ phase (tetragonal lattice, $\alpha = \beta = \gamma = 90°$):

$$\mathbf{a} = \mathbf{b} = \frac{\sqrt{17}d_{410}+3\sqrt{2}d_{330}}{2} \quad \textbf{(S1)}$$

$$\mathbf{c} = 2d_{002} \quad \textbf{(S2)}$$

Diameter of the supramolecular micelle (tetragonal lattice):

$$\mathbf{D}_{sphere} = 2\left(\frac{abc}{40\pi}\right)^{\frac{1}{3}} \quad \textbf{(S3)}$$

**Table S3** List of the DSC parameters of pure **D2** and **Py2/D2** mixtures.

| | **D2** | $X_{Py_2} = 0.1$ | $X_{Py_2} = 0.2$ | $X_{Py_2} = 0.3$ | $X_{Py_2} = 0.4$ |
|---|---|---|---|---|---|
| $T_i$ (°C) | 66.5 | 64.9 | 64.2 | 62.2 | 62.7 |
| $\triangle_i$H (kJ mol$^{-1}$) | 2.2 | 1.3 | 1.2 | 0.52 | 0.54 |
| $\triangle_i$S (J mol$^{-1}$ K$^{-1}$) | 6.5 | 3.7 | 3.4 | 1.6 | 1.6 |

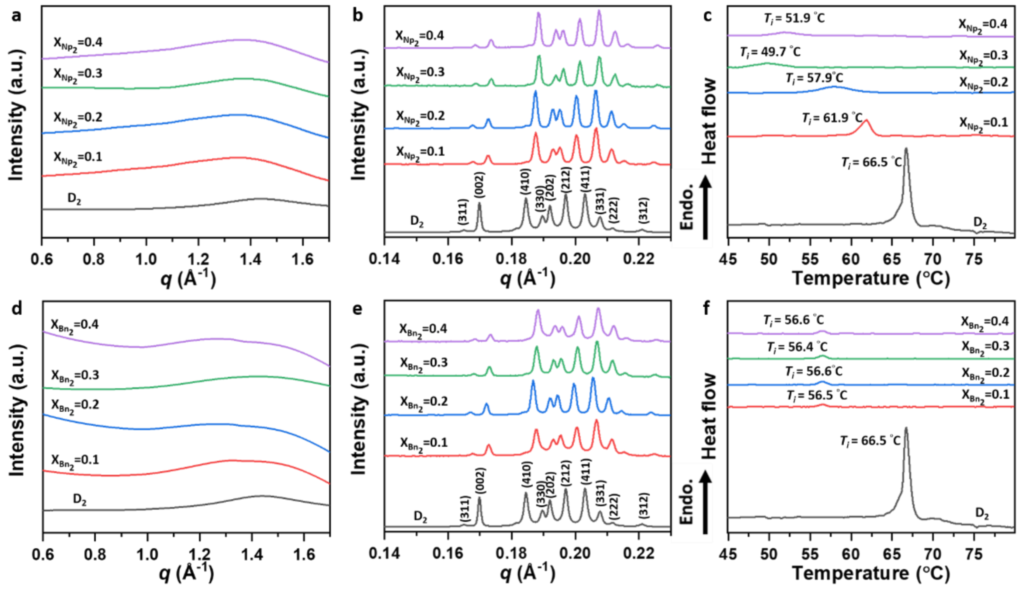


**Fig. S18** (a) WAXS and (b) SAXS patterns, and (c) DSC thermograms of **Np2/D2** mixtures. (d) WAXS and (e) SAXS patterns, and (f) DSC thermograms of **Bn2/D2** mixtures.

**Table S4** List of the $q$ ratio of the $\sigma$ phase in the literature ($q_{lit}$)[1] and the experimental $q_{exp}$ for the FK σ phase of the **$Np_2$/$D_2$** mixtures.

| Miller index | $X_{Np_2}$ = 0.1 | $X_{Np_2}$ = 0.2 | $X_{Np_2}$ = 0.3 | $X_{Np_2}$ = 0.4 | $X_{Np_2}$ = 0.1 | $X_{Np_2}$ = 0.2 | $X_{Np_2}$ = 0.3 | $X_{Np_2}$ = 0.4 | $q_{lit}$ ratio |
|---|---|---|---|---|---|---|---|---|---|
| | $q_{exp}$ | | | | $q_{exp}$ ratio | | | | |
| (310) | 0.1437 | 0.1437 | 0.1441 | 0.1445 | 1.00 | 1.00 | 1.00 | 1.00 | 1.00 |
| (311) | 0.1676 | 0.1676 | 0.1688 | 0.1684 | 1.19 | 1.19 | 1.17 | 1.17 | 1.17 |
| (002) | 0.1724 | 0.1728 | 0.1736 | 0.1736 | 1.20 | 1.20 | 1.20 | 1.20 | 1.20 |
| (410) | 0.1875 | 0.1875 | 0.1887 | 0.1883 | 1.30 | 1.30 | 1.31 | 1.30 | 1.30 |
| (330) | 0.1931 | 0.1931 | 0.1939 | 0.1939 | 1.34 | 1.34 | 1.34 | 1.34 | 1.34 |
| (202) | 0.1951 | 0.1951 | 0.1963 | 0.1963 | 1.36 | 1.36 | 1.36 | 1.36 | 1.36 |
| (212) | 0.2002 | 0.2002 | 0.2014 | 0.2014 | 1.39 | 1.39 | 1.40 | 1.39 | 1.39 |
| (411) | 0.2066 | 0.2062 | 0.2074 | 0.2074 | 1.44 | 1.43 | 1.44 | 1.44 | 1.44 |
| (331) | 0.2114 | 0.2114 | 0.2126 | 0.2126 | 1.47 | 1.47 | 1.47 | 1.47 | 1.47 |
| (222) | 0.2154 | 0.2154 | 0.2166 | 0.2166 | 1.50 | 1.50 | 1.50 | 1.50 | 1.50 |
| (312) | 0.2245 | 0.2245 | 0.2257 | 0.2261 | 1.56 | 1.56 | 1.57 | 1.56 | 1.56 |

**Table S5** List of the lattice parameters (a, b, c) and the diameter of supramolecular micelles ($D_{sphere}$) for the FK σ phase of the **$Np_2$/$D_2$** mixtures.

| | $X_{Np_2}$ = 0.1 | $X_{Np_2}$ = 0.2 | $X_{Np_2}$ = 0.3 | $X_{Np_2}$ = 0.4 |
|---|---|---|---|---|
| a (Å) | 138.0 | 138.0 | 137.3 | 137.3 |
| b (Å) | 138.0 | 138.0 | 137.3 | 137.3 |
| c (Å) | 72.8 | 72.7 | 72.3 | 72.3 |
| $D_{sphere}$ (Å) | 44.5 | 44.5 | 44.3 | 44.3 |

**Table S6** List of the DSC parameters of pure **$D_2$** and **$Np_2$/$D_2$** mixtures.

| | **$D_2$** | $X_{Np_2}$ = 0.1 | $X_{Np_2}$ = 0.2 | $X_{Np_2}$ = 0.3 | $X_{Np_2}$ = 0.4 |
|---|---|---|---|---|---|
| $T_i$ (°C) | 66.5 | 61.9 | 57.9 | 49.7 | 51.9 |
| $\triangle_i H$ (kJ mol$^{-1}$) | 2.2 | 0.71 | 0.69 | 0.50 | 0.36 |
| $\triangle_i S$ (J mol$^{-1}$ K$^{-1}$) | 6.5 | 2.1 | 2.1 | 1.5 | 1.1 |

**Table S7:** List of the $q$ ratio of the $\sigma$ phase in the literature[4] ($q_{lit}$) and the experimental $q_{exp}$ for the FK σ phase of the **Bn2/D2** mixtures.

| Miller index | $X_{Bn_2}$ = 0.1 | $X_{Bn_2}$ = 0.2 | $X_{Bn_2}$ = 0.3 | $X_{Bn_2}$ = 0.4 | $X_{Bn_2}$ = 0.1 | $X_{Bn_2}$ = 0.2 | $X_{Bn_2}$ = 0.3 | $X_{Bn_2}$ = 0.4 | $q_{lit}$ ratio |
|---|---|---|---|---|---|---|---|---|---|
| | $q_{exp}$ | | | | $q_{exp}$ ratio | | | | |
| (310) | 0.1425 | 0.1429 | 0.1429 | 0.1445 | 1.00 | 1.00 | 1.00 | 1.00 | 1.00 |
| (311) | 0.1676 | 0.1668 | 0.1680 | 0.1684 | 1.18 | 1.17 | 1.18 | 1.17 | 1.17 |
| (002) | 0.1727 | 0.1720 | 0.1728 | 0.1732 | 1.21 | 1.20 | 1.21 | 1.20 | 1.20 |
| (410) | 0.1879 | 0.1867 | 0.1879 | 0.1883 | 1.32 | 1.31 | 1.31 | 1.30 | 1.30 |
| (330) | 0.1931 | 0.1919 | 0.1931 | 0.1935 | 1.35 | 1.34 | 1.35 | 1.34 | 1.34 |
| (202) | 0.1955 | 0.1943 | 0.1955 | 0.1959 | 1.37 | 1.36 | 1.37 | 1.36 | 1.36 |
| (212) | 0.2006 | 0.1994 | 0.2006 | 0.2010 | 1.40 | 1.39 | 1.40 | 1.39 | 1.39 |
| (411) | 0.2066 | 0.2054 | 0.2066 | 0.2070 | 1.45 | 1.44 | 1.44 | 1.43 | 1.44 |
| (331) | 0.2114 | 0.2106 | 0.2118 | 0.2122 | 1.48 | 1.47 | 1.48 | 1.47 | 1.47 |
| (222) | 0.2154 | 0.2146 | 0.2158 | 0.2158 | 1.51 | 1.50 | 1.51 | 1.49 | 1.50 |
| (312) | 0.2249 | 0.2237 | 0.2253 | 0.2253 | 1.58 | 1.56 | 1.58 | 1.56 | 1.56 |

**Table S8:** List of the lattice parameters (a, b, c) and the diameter of supramolecular micelles ($D_{sphere}$) for the FK σ phase of the **Bn2/D2** mixtures.

| | $X_{Bn_2}$ = 0.1 | $X_{Bn_2}$ = 0.2 | $X_{Bn_2}$ = 0.3 | $X_{Bn_2}$ = 0.4 |
|---|---|---|---|---|
| a (Å) | 137.9 | 138.6 | 137.9 | 137.6 |
| b (Å) | 137.9 | 138.6 | 137.9 | 137.6 |
| c (Å) | 72.7 | 72.7 | 72.5 | 72.5 |
| $D_{sphere}$ (Å) | 44.5 | 44.6 | 44.5 | 44.4 |

**Table S9:** List of the DSC parameters of pure **D2** and **Bn2/D2** mixtures.

| | **D2** | $X_{Bn_2}$ = 0.1 | $X_{Bn_2}$ = 0.2 | $X_{Bn_2}$ = 0.3 | $X_{Bn_2}$ = 0.4 |
|---|---|---|---|---|---|
| $T_i$ (°C) | 66.5 | 56.5 | 56.6 | 56.4 | 56.6 |
| $\Delta_i H$ (kJ mol$^{-1}$) | 2.2 | 0.061 | 0.062 | 0.062 | 0.061 |
| $\Delta_i S$ (J mol$^{-1}$ K$^{-1}$) | 6.5 | 0.2 | 0.2 | 0.2 | 0.2 |

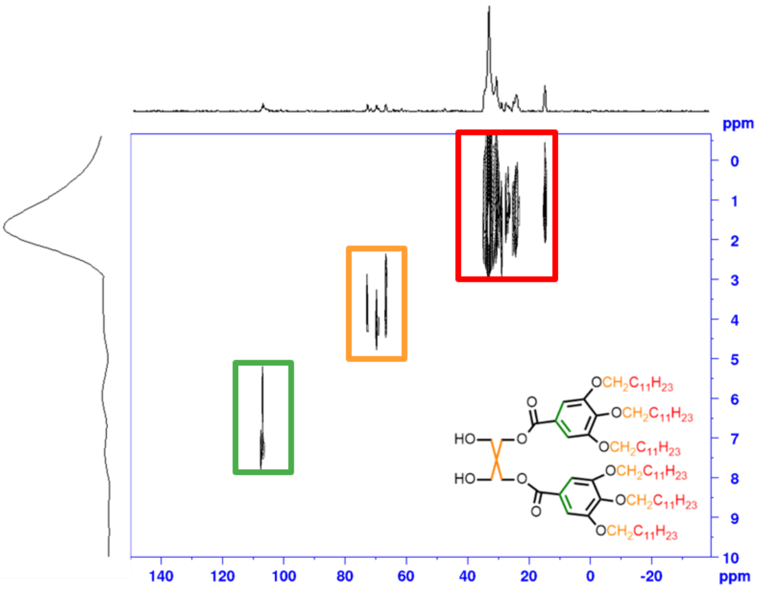


**Fig. S19** The ssNMR HETCOR spectra of **D2** in a Lam/σ coexistence state. ($t_c = 0.2$ ms)

The HETCOR spectra of **D2** show a pronounced dependence on the thermal and phase history of the sample. When **D2** exists in a Lamellar/σ (Lam/σ) coexistence state, the heterogeneous local environments combined with reduced molecular mobility allow efficient $^{1}H$-$^{13}C$ dipolar recoupling under CP conditions, enabling the detection of all characteristic resonances of the molecule. In this state, the O–$CH_2$ units resonate in the downfield region (δC ≈ 65–75 ppm), the pentaerythritol core appears at δC = 50–65 ppm, and the aromatic units give rise to signals in the range of δC = 100–120 ppm. Upon heating to the isotropic phase followed by cooling to the Frank–Kasper (FK) σ phase, the system undergoes a Lam → σ phase transition and becomes significantly more mobile.[3] In the resulting pure σ phase, fast local motions markedly attenuate the $^{1}H$–$^{13}C$ dipolar couplings, rendering CP transfer inefficient for the more dynamic molecular segments. As a consequence, only signals from the relatively abundant and less mobile dodecyl chains remain observable, whereas the resonances of the O–$CH_2$ units and the pentaerythritol core (orange box), as well as those of the aromatic units (green box), are no longer detected, as illustrated in Fig. 2a.

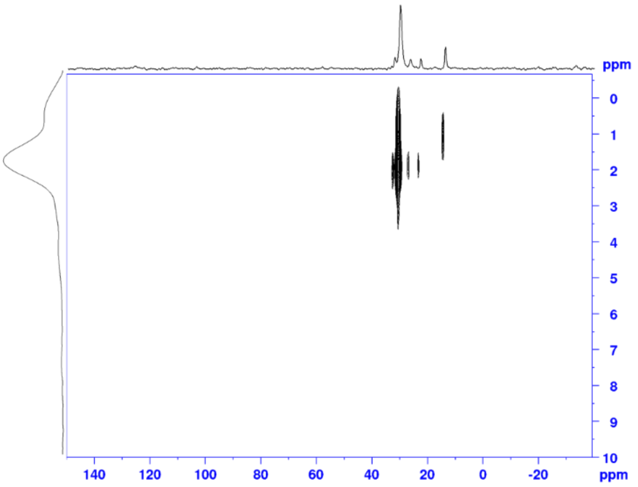


**Fig. S20** The ssNMR HETCOR spectra of $\boldsymbol{X_{Py_2}} = 0.1$ mixture. ($t_c = 5$ ms)

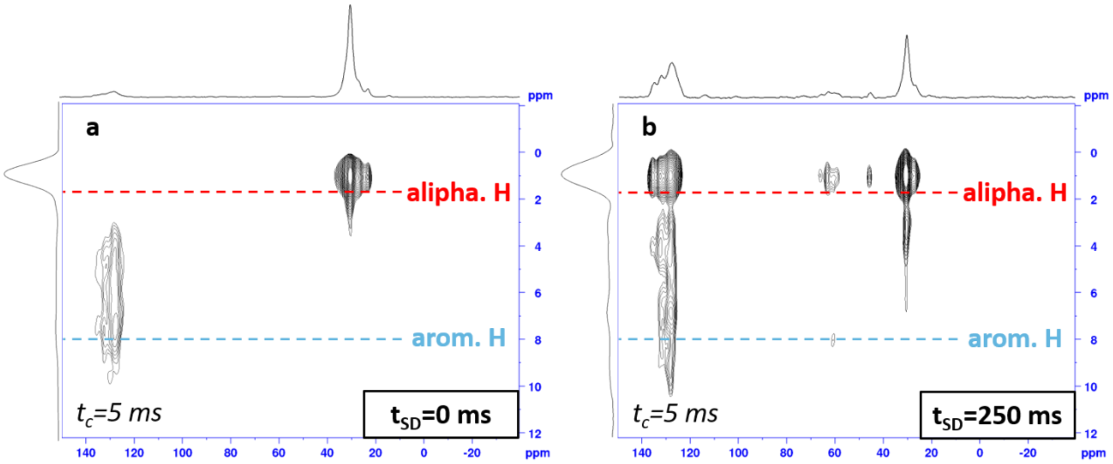


**Fig. S21** (a) **Np2/D2** mixture with $\boldsymbol{X_{Np_2}} = 0.2$ and (b) proton spin-diffusion-incorporated HETCOR spectra of the$\boldsymbol{X_{Np_2}} = 0.2$ mixture at $t_{SD} = 250$ ms.

## 3. Characterization of the An@σ($Ar_2$)

**Table S10:** List of the *q* value for the FK σ phase of the **An**-loaded mixtures.

| Miller index | **An@σ** | **An@σ($Py_2$)** | **An@σ($Np_2$)** | **An@σ($Bn_2$)** |
|---|---|---|---|---|
| (311) | 0.1732 | 0.1667 | 0.1679 | 0.1687 |
| (002) | 0.1786 | 0.1715 | 0.1731 | 0.1735 |
| (410) | 0.1936 | 0.1862 | 0.1879 | 0.1883 |
| (330) | 0.1993 | 0.1919 | 0.1931 | 0.1938 |
| (202) | 0.2020 | 0.1938 | 0.1955 | 0.1962 |
| (212) | 0.2072 | 0.1990 | 0.2006 | 0.2014 |
| (411) | 0.2133 | 0.2050 | 0.2066 | 0.2074 |
| (331) | 0.2183 | 0.2102 | 0.2114 | 0.2126 |
| (222) | 0.2226 | 0.2138 | 0.2158 | 0.2162 |
| (312) | 0.2323 | 0.2230 | 0.2254 | 0.2261 |

**Table S11:** List of the lattice parameters (a, b, c) and the diameter of supramolecular micelles ($D_{sphere}$) for the FK σ phase of the **An**-loaded mixtures.

| | **An@σ** | **An@σ($Py_2$)** | **An@σ($Np_2$)** | **An@σ($Bn_2$)** |
|---|---|---|---|---|
| a (Å) | 133.7 | 139.0 | 137.9 | 137.5 |
| b (Å) | 133.7 | 139.0 | 137.9 | 137.5 |
| c (Å) | 70.3 | 73.2 | 72.6 | 72.4 |
| $D_{sphere}$ (Å) | 44.1 | 44.8 | 44.5 | 44.3 |

**Table S12:** The lattice shrinkage of **An**-incorporated systems along each axis (compare with systems without **An**)

| | **An@σ** | **An@σ(Py2)** | **An@σ(Np2)** | **An@σ(Bn2)** |
|---|---|---|---|---|
| $S_a$ (%) | 5.2 | 0.2 | 0 | 1.0 |
| $S_b$ (%) | 5.2 | 0.2 | 0 | 1.0 |
| $S_c$ (%) | 5.2 | 1.0 | 0.1 | 0.8 |

**Table S12** reports the lattice shrinkage of **An**-incorporated systems along each crystallographic axis relative to the guest-free counterparts, calculated using:

$$\boldsymbol{S_X}(\%) = \frac{\boldsymbol{X_i} - \boldsymbol{X_f}}{\boldsymbol{X_f}}$$

where X denotes the lattice parameter along a given axis (Å), and the subscripts refer to the states before and after guest incorporation. Conventional micelles show notable contraction along all axes, with **An@σ** exhibiting the largest overall shrinkage, indicating substantial perturbation of the micellar packing. In contrast, **σ(Ar2)** micelles display only minor lattice changes, suggesting that the concave domains effectively confine guest molecules and prevent extensive global distortion, preserving the underlying FK σ architecture.

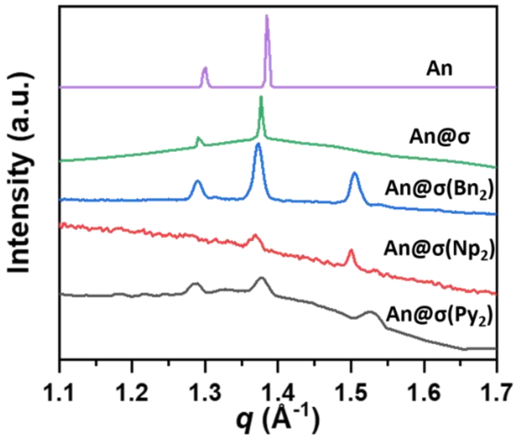


**Fig. S22** WAXS patterns of **An@σ(Ar2)** and **An@σ** systems. Note: The $X_{Ar_2}$ is equal to 0.2 and the molar ratio of the encapsulated **An** and **Ar2** is 2:1 in the **An@σ(Ar2)** samples.

WAXS profiles in Fig. S22 further highlight the distinction: a new peak at $q$ = 1.50 Å$^{-1}$ (d = 4.19 Å) emerges in concaved micelles, indicative of concavity-stabilized interactions, while **An** in **Py2**- and **Bn2**-based assemblies readily phase-separates.

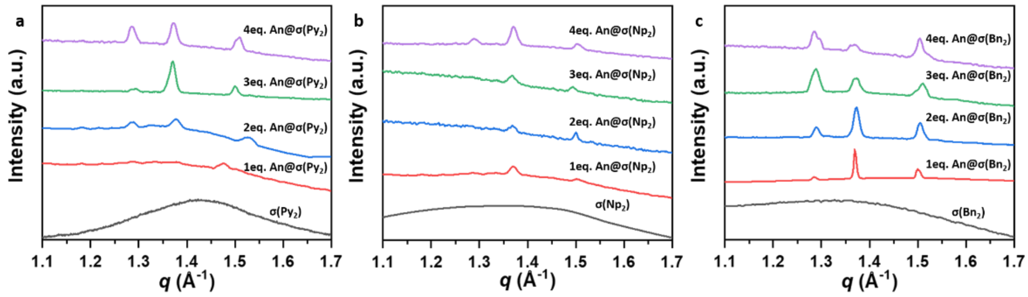


**Fig. S23** The WAXS patterns of **An@σ(Ar2)** with different amount **An** introduced into (a) **σ(Py2)**, (b) **σ(Np2)**, and (c) **σ(Bn2)**. Note: the equivalence (eq.) of **An** indicate the relative eq. to the **Ar2** in the **σ(Ar2)** phase with $X_{Ar_2}$ = 0.2.

According to Fig. S23, all **σ(Ar2)** variants except **σ(Bn2)** are capable of encapsulating at least 1 eq. of **An**. At lower **An** loadings, a distinct diffraction peak at $q$ = 1.50 Å$^{-1}$

emerges first, whereas the characteristic crystalline **An** peaks only appear at higher equivalents. This behavior suggests that the micellar cavities initially engage guest molecules via specific π–π intermolecular interactions, leading to the formation of micellar complexes. Crystallization of excess **An** occurs only once the encapsulation capacity is exceeded. The efficiency of complex formation is dictated by both π–π interaction strength and cavity depth. **Bn$_2$**, possessing the smallest raft-inspired building block, reaches saturation at approximately 1 eq.. **Py$_2$**, with the largest building block, accommodates slightly more **An** due to stronger π–π interactions. Notably, **Np$_2$** offers the most effective balance between interaction strength and geometric compatibility, resulting in optimal guest encapsulation.

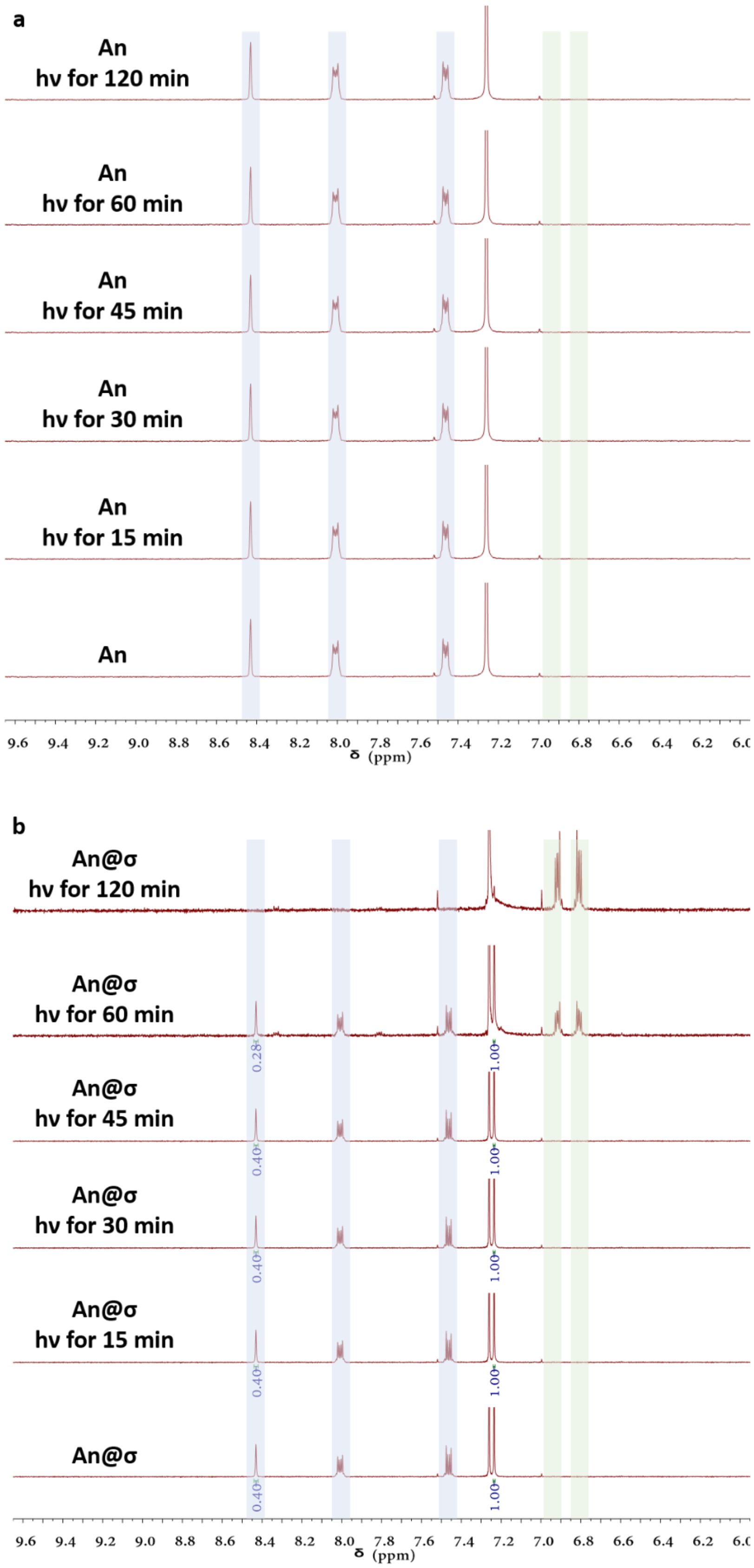
a
An
hv for 120 min
An
hv for 60 min
An
hv for 45 min
An
hv for 30 min
An
hv for 15 min
An
9.6 9.4 9.2 9.0 8.8 8.6 8.4 8.2 8.0 7.8 7.6 7.4 7.2 7.0 6.8 6.6 6.4 6.2 6.0
δ (ppm)
b
An@σ
hv for 120 min
An@σ
hv for 60 min
0.28
1.00
An@σ
hv for 45 min
0.40
1.00
An@σ
hv for 30 min
0.40
1.00
An@σ
hv for 15 min
0.40
1.00
An@σ
0.40
1.00
9.6 9.4 9.2 9.0 8.8 8.6 8.4 8.2 8.0 7.8 7.6 7.4 7.2 7.0 6.8 6.6 6.4 6.2 6.0
δ (ppm)

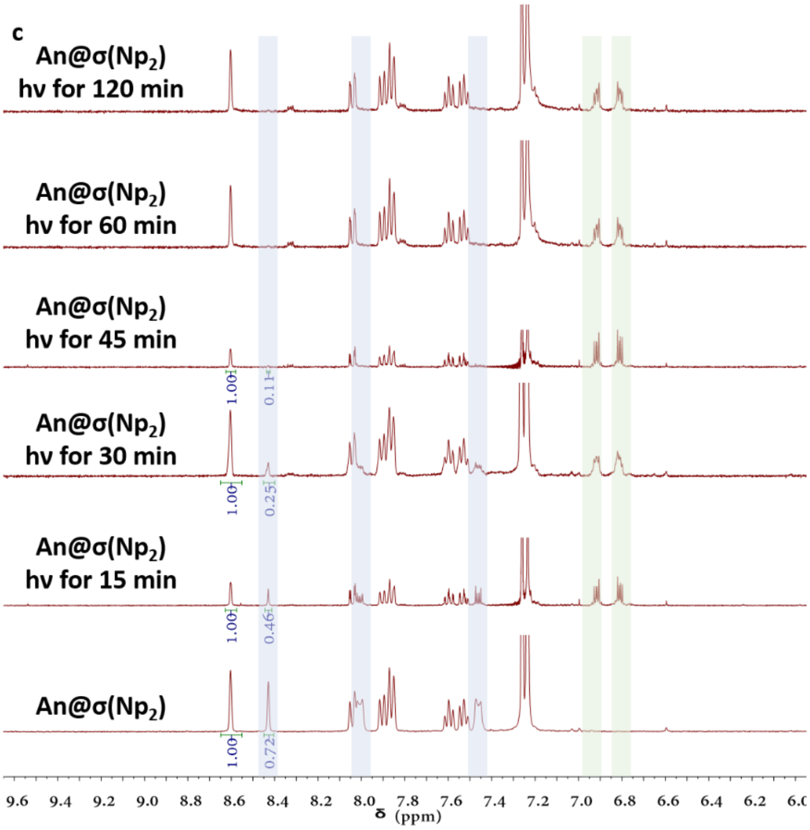


**Fig. S24** Time-dependent ex-situ $^1$H NMR local spectra for the photodimerization process of (a) crystalline **An**, (b) **An@σ** and **(c) An@σ(Np2)** (400 MHz in $CDCl_3$) Note: (1) Proton doublet signals at about 7.24 ppm for **D2** and signal at about 8.61 ppm for **Np2** have been used as internal standard to trace the **An** singlet signal at about 8.43 ppm. (2) The signals highlighted with blue and green background in the figure correspond to the $^1$H NMR peaks of **An** and dianthracene, respectively.

# Reference


1. T. Jun, H. Park, S. Jeon, S. Jo, H. Ahn, W.-D. Jang, B. Lee and D. Y. Ryu, *J. Am. Chem. Soc.*, 2021, **143**, 17548-17556.

2. M. N. Holerca, D. Sahoo, B. E. Partridge, M. Peterca, X. Zeng, G. Ungar and V. Percec, *J. Am. Chem. Soc.*, 2018, **140**, 16941-16947.

3. C.-L. Wang, W.-T. Chuang, M.-T. Lee, Y.-R. Wang, S.-Y. Chen, H.-J. Huang, S.-Y. Liu, J.-M. Lin, C.-Y. Chen and Y.-C. Lee, *ACS Appl. Mater. Interfaces*, 2025, **17**, 31403-31410.
